# Antimicrobial resistance in Ecuador: A One Health situational analysis of governance, infrastructure, and equity gaps

**DOI:** 10.1371/journal.pgph.0005308

**Published:** 2026-08-21

**Authors:** Richar Rodriguez-Hidalgo, Linette Jácome, Diana Paz, Arne Ruckert, Raphael Aguiar, Mayumi Duarte Wakimoto, Phaedra Henley, Mary Wiktorowicz

**Affiliations:** 1 Facultad de Medicina Veterinaria y Zootecnia, Universidad Central del Ecuador, Quito, Ecuador; 2 Instituto de Investigación en Zoonosis, Universidad Central del Ecuador, Quito, Ecuador; 3 Global Strategy Lab, York University, Toronto, Ontario, Canada; 4 School of Health Policy and Management, York University, Toronto, Ontario, Canada; 5 Instituto Nacional de Infectologia Evandro Chagas, Fundação Oswaldo Cruz, Rio de Janeiro, Brazil; 6 Center for One Health, University of Global Health Equity, Butaro, Rwanda; 7 School of Global Health, York University, Toronto, Ontario, Canada; University of Oxford, UNITED KINGDOM OF GREAT BRITAIN AND NORTHERN IRELAND

## Abstract

Antimicrobial resistance (AMR) represents a growing global public health threat, undermining therapeutic efficacy and disproportionately affecting vulnerable populations. In Ecuador, the situation is exacerbated by weak surveillance systems and fragmentation among the human, animal, and environmental health sectors. This study analyzes current national approaches that address AMR in Ecuador from a One Health (OH) perspective, with an emphasis on governance, public policy, health infrastructure, and equity. A qualitative approach was employed combining document review, scientific literature analysis, and semi-structured interviews with key informants (KIs) engaged across all OH sectors. Findings reveal structural barriers, including lack of institutionalization of the OH strategy, limited funding for the health system, weak intersectoral coordination, low community awareness, and the absence of equity-oriented policies. In conclusion, the results highlight the urgent need to strengthen the governance of antimicrobial stewardship in Ecuador through strategies that are contextually adapted, aligned with international recommendations, and grounded in a comprehensive multisectoral approach.

## Introduction

Antimicrobial resistance (AMR) constitutes a critical threat to global health [1], undermining decades of progress in the treatment of infectious diseases and contributing to increased morbidity, mortality, and healthcare costs worldwide [2–5]. Estimates suggest that AMR caused more than 1.27 million deaths in 2019, with the most severe impacts observed in low- and middle-income countries [6,7]. In Ecuador, several factors including socioeconomic inequality, limited and inefficient health infrastructure, self-medication, and the inappropriate use of antimicrobials in both human and animal health exacerbate the situation [8–10]. These conditions contribute to selective pressure [7], whereby widespread and inappropriate antimicrobial use favors the survival and proliferation of resistant microorganisms while eliminating susceptible ones, thereby accelerating the emergence and spread of AMR.

The One Health (OH) approach recognizes the interdependence of human, animal, and environmental health and promotes integrated responses to complex threats such as AMR, which transcend the boundaries of traditional health sectors [11]. This includes raising awareness and improving understanding through effective communication, education, and training; strengthening the evidence base through systematic surveillance and research; reducing infection incidence through improved sanitation, hygiene, and preventive measures; promoting the prudent use of antimicrobials in both human and animal health; and securing sustained investment in the development of new medicines, diagnostics, and vaccines. In this study, the OH approach was used as an analytical and governance-oriented framework to examine the interactions, coordination mechanisms, and implementation challenges across the human, animal, and environmental health sectors in relation to AMR governance in Ecuador [12].

Ecuador adopted the National Plan for the Prevention and Control of Antimicrobial Resistance (2019–2023), aligned with the Global Action Plan on AMR and the OH approach. The Plan aimed to strengthen multisectoral governance, improve epidemiological surveillance and laboratory capacity, promote the rational use of antimicrobials in human and animal health, enhance infection prevention and control, and foster education, research, and public awareness. Its implementation relied on interinstitutional coordination among health, agriculture, and environmental authorities and was supported by a national AMR committee intended to oversee planning, coordination, and cross-sectoral monitoring [10]. Effective implementation of the Plan required sustained political commitment, institutional continuity, adequate technical capacity, and sufficient financial resources.

In Ecuador, the National AMR Committee was proposed in 2017 and legally formalized in 2020, as identified through document analysis. However, implementation of the national plan has faced multiple structural barriers. Policy documents and key informant (KI) interviews identified concerns related to the limited institutionalization of the National AMR Committee, weak intersectoral coordination, bureaucratic delays, recurrent financial, technical, and human resource constraints, and limited political prioritization of AMR within the national agenda [10]. Findings from policy documents and KI interviews further suggested that political discontinuity and institutional fragmentation may have hindered sustained multisectoral collaboration. These challenges were further exacerbated during the COVID-19 pandemic, which generated additional operational and financial pressures on AMR surveillance and response systems [13]. According to secondary literature on health system governance in Ecuador, the political and economic transition initiated after 2017 was associated with austerity-oriented fiscal policies, reductions in public spending, and institutional restructuring processes that may have affected health system capacities and governance continuity [14]. Previous analyses have also suggested that these policy and fiscal changes were associated with reduced access to public services, pressures on health infrastructure, and operational constraints affecting health system responsiveness, particularly during the COVID-19 pandemic [15].

By analyzing the AMR landscape in Ecuador from a One Health (OH) perspective, this study examines the governance and implementation of antimicrobial stewardship policies, focusing on key dimensions such as public policy development, institutional infrastructure, technical surveillance capacity, and equity considerations. In particular, the study identifies structural, institutional, and operational barriers, as well as enabling factors, that shape the implementation of the national AMR response within the OH framework, including persistent challenges in intersectoral coordination. The study also highlights opportunities to strengthen collaboration, institutional communication, and community and stakeholder engagement mechanisms. Understanding these governance dynamics is essential for the development of sustainable and contextually adapted strategies to strengthen AMR response capacities in Ecuador and similar settings.

This study does not aim to assess AMR prevalence, microbiological patterns, or epidemiological trends associated with antimicrobial resistance. Rather, it seeks to contribute to local and regional discussions on AMR governance in Latin America by emphasizing the need for more robust, equitable, and interculturally sensitive public policies framed within the OH approach. Based on the analysis of the Ecuadorian case, the study proposes a context-specific action framework intended to support decision-making processes and inform governance strengthening efforts in other countries across the region. This framework is further developed in the Discussion subsection entitled “A context-specific action framework for strengthening AMR governance in Ecuador under a One Health approach”.

## Materials and methods

### Ethics statement

This study received ethical approval from the Ethics Committee on Human Research of the Universidad Central del Ecuador (CEISH Code “006-DOC-FMVZ-2023”). All participants were prospectively recruited and provided written informed consent prior to participation. For in-person interviews, signed consent forms were obtained immediately before data collection. For participants interviewed virtually, consent forms were sent in advance by email, signed electronically, and returned before the interview was conducted.

A qualitative research approach was adopted to conduct an environmental scan of governance, infrastructure, public policy, and equity dimensions related to AMR in Ecuador within a One Health (OH) framework. The study was conceptually informed by the OH approach as an integrated governance framework emphasizing multisectoral coordination, interdependence among human, animal, and environmental health systems, and the need for collaborative institutional responses to complex public health challenges such as AMR [12]. This qualitative policy and governance analysis combined document analysis of regulatory, institutional, and policy sources from both the public and private sectors with semi-structured interviews conducted with key stakeholders across the human, animal, and environmental health sectors. The study design was intended to examine institutional arrangements, intersectoral coordination mechanisms, and governance gaps shaping AMR response implementation, rather than to assess epidemiological trends, microbiological patterns, or AMR prevalence.

### Study design and setting

A case study of Ecuador was conducted within a broader comparative multi-country study involving Canada, Brazil, Rwanda, and Ecuador. These countries were selected to represent diverse geographical, institutional, and socioeconomic contexts for examining governance and implementation challenges related to antimicrobial resistance under the OH approach. The broader study aimed to identify barriers, gaps, and opportunities for strengthening AMR governance across different multisectoral and policy environments. Two qualitative methods were employed to conduct the environmental scan and institutional governance analysis: (1) document analysis and (2) semi-structured interviews with key informants (KIs). The document analysis followed the thematic approach described by Braun and Clarke (2006) [16], applying systematic coding procedures to identify patterns related to AMR governance structures, institutional roles, policy implementation processes, and intersectoral coordination mechanisms. Relevant policy documents and regulatory instruments were imported into NVivo software to support data organization, coding consistency, thematic analysis, and triangulation with interview findings across the predefined analytical domains. Semi-structured interviews were conducted using an interview guide with predefined thematic areas while allowing flexibility to explore participants’ experiences, perceptions, and institutional perspectives in greater depth through follow-up questions [16,17].

### Data sources and data collection

#### Document analysis.

The document analysis was based on a systematic search strategy applied to institutional, academic, legal, and policy-related sources in both Spanish and English. Searches were conducted using Google, Google Scholar, and PubMed, in addition to the official websites of the Ministry of Public Health (MSP), Ministry of Agriculture and Livestock (MAG), Ministry of Environment, Water and Ecological Transition (MAATE), National Agency for Health Regulation and Control (ARCSA), and relevant international organizations involved in AMR governance and OH initiatives.

Search terms included “AMR”, “antimicrobial resistance,” “antibiotic” OR “antimicrobial,” “AMR Action Plan” OR “National AMR Plan,” “antimicrobial resistance in Ecuador” OR “Antimicrobial use in Ecuador,” “One Health,” and their Spanish equivalents (“Una Salud” or “Una Sola Salud”). Documents published between 2020 and 2024 were included if they were directly relevant to the Ecuadorian context and addressed AMR governance, surveillance, public policy, or One Health-related issues. Eligible materials included laws, regulations, executive decrees, technical reports, public policies, sectoral strategies, and national and international scientific literature.

**Fig 1:**
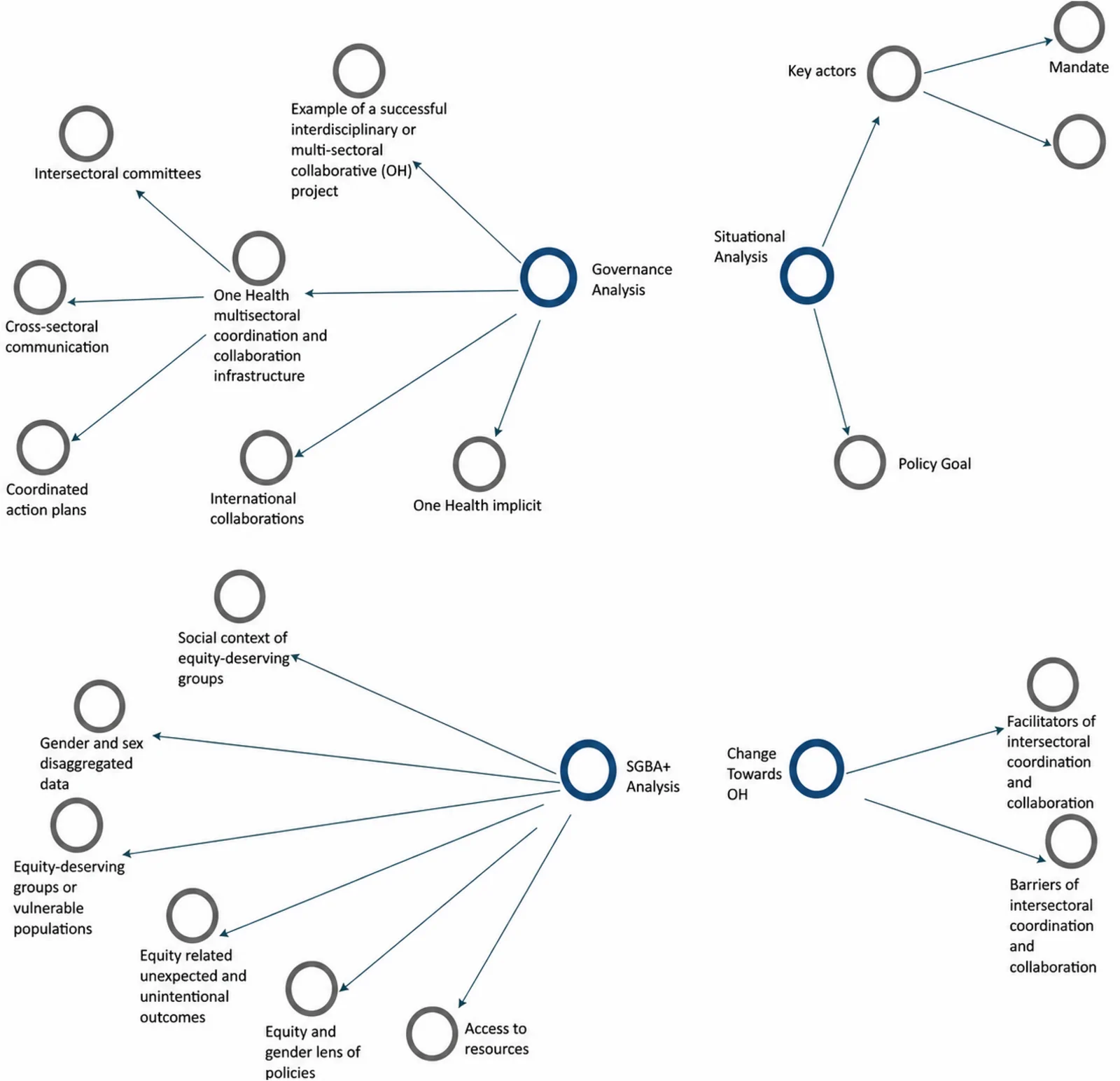
Thematic coding structure used for the NVivo analysis. Note. Diagram illustrating the thematic nodes (blue spheres) and sub-nodes (white spheres) applied during the qualitative coding process in NVivo software.

A total of 117 relevant documents were retained following a systematic screening and eligibility assessment process. Initially, 499 documents related to global public health and United Nations priority challenges were identified, of which 240 broadly addressed AMR-related issues. Of these, 137 focused specifically on AMR in Ecuador, and 117 met the predefined inclusion criteria for in-depth analysis. All eligible documents were imported into NVivo 13 software (version 1.7.2) and analyzed using thematic coding procedures (Fig 1), based on a structured codebook shared across the four participating countries. The main analytical domains included governance, infrastructure, public policy, and equity dimensions related to AMR and OH implementation [16].

#### Semi-structured interviews.

A total of 23 semi-structured interviews were conducted with KIs selected through purposive sampling based on their direct experience in the design, implementation, monitoring, or governance of AMR-related policies and strategies. Participants represented human, animal, and environmental health sectors, as well as academia, civil society, and professional associations [18] (Table 1). Eligibility criteria included current or recent involvement in AMR-related governance, policy development, surveillance, or implementation activities at national or sectoral levels.

**Table 1:** Background of interview participants.

One Health domain/ Sector	Institutional affiliation/ Area of expertise	Number of participants	Percentage
Academia	Universities (public and private), AMR research and training	9	39.13
Civil society/ Professional associations	Professional associations and civil society organizations working on AMR	6	26.09
Human health	Ministry of Public Health (MSP) and affiliated agencies^a^ (INSPI, ARCSA)	2	8.70
Animal health	Ministry of Agriculture and Livestock (MAG) and affiliated veterinary services	3	13.04
Private sector	Private institutions involved in health or veterinary services	2	8.70
Environmental health	Ministry of Environmental, Water, and Ecological Transition (MAATE); climate change and AMR	1	4.35
**Total**		**23**	**100.00**

^a^Affiliated agencies refer to national public institutions and regulatory bodies operating under the respective ministries, including entities responsible for surveillance, regulation, and technical implementation (e.g., INSPI and ARCSA).

The interview guide was developed through expert consultation and organized around predefined thematic domains, including governance structures, intersectoral coordination mechanisms, implementation of the National AMR Plan, infrastructure gaps, and equity-oriented approaches. Recruitment and data collection were conducted between March 1 and June 15, 2024.

The interviews had an average duration of approximately 45 minutes and were conducted using a hybrid format (in-person and virtual), depending on participants’ availability. All interviews were audio-recorded with participants’ consent, and no minors were involved in the study. Interview transcripts were subsequently analyzed through thematic coding in NVivo by two researchers (LJ and DP) who independently reviewed the data and collaboratively agreed on the final thematic structure and interpretation of findings. Areas of disagreement were discussed until consensus was reached. Findings from the interviews were triangulated with results obtained through document analysis to strengthen analytical consistency and interpretative validity.

#### Data management.

All documents and interview transcripts were stored on password-protected institutional devices with access restricted to members of the research team. Interview data were de-identified prior to analysis to protect participant confidentiality. Identifiable information was removed from transcripts and excluded from all analytical outputs and reporting processes. NVivo software was used to support the organization, management, coding, and thematic analysis of qualitative data.

#### Analytical framework and data analysis.

The analytical framework was developed to operationalize the OH approach within a qualitative governance-oriented analysis, emphasizing the interdependence of human, animal, and environmental health systems, as well as the importance of intersectoral coordination, institutional interactions, surveillance capacities, implementation processes, and equity dimensions relevant to AMR governance [12]. This framework guided the examination of governance structures, stakeholder´s interactions, operational barriers, and multisectoral implementation challenges affecting AMR response in Ecuador, drawing on contemporary OH governance approaches described in the literature [12,19–23].

Selection of the analytical domains and stakeholder-related dimensions was informed by the study objectives and by contemporary OH governance approaches emphasizing institutional coordination, multisectoral participation, implementation processes, and governance interactions relevant to AMR response. Particular attention was given to stakeholder roles, institutional responsibilities, levels of involvement, intersectoral communication mechanisms, and operational capacities across the human, animal and environmental health sectors.

Based on this OH governance-oriented analytical perspective, four analytical domains were defined as the interpretative framework guiding this study:

1. **Institutional governance analysis**: assessment of institutional arrangements, coordination mechanisms, and multisectoral collaboration processes within the OH framework.
2. **Situational analysis**: identification of stakeholder roles, institutional positions, priorities, and levels of involvement in AMR-related governance processes.
3. **Transition toward One Health**: examination of facilitators, barriers, and operational challenges associated with intersectoral adoption and implementation of the OH approach.
4. **Equity analysis using a Gender-Based Analysis Plus (GBA+) lens**: evaluation of how AMR-related policies and governance processes address gender equity, access to services, and impacts on vulnerable populations. The GBA+ framework was used as an analytical tool to examine how gender and other intersecting social determinants may influence exposure, access to services, participation, and vulnerability within AMR-related governance and policy processes [22,23].

These analytical domains were operationalized through thematic nodes and sub-nodes coded in NVivo and organized within a thematic framework that facilitated the integration and triangulation of findings from document analysis and qualitative interview data [24] (Fig 1). Detailed results of the thematic coding of AMR-related documents are presented in S1 Table, while the complete NVivo coding structure and node hierarchy are provided as S1 Text. Summarized and anonymized findings from KIs are presented in S2 Table.

The NVivo-based analysis enabled the systematic coding and organization of 117 documents from multiple institutional, academic, legal, and policy-related sources. The coding process generated 742 coding instances across documents and 1,657 thematic references corresponding to coded text segments and analytical excerpts. The analyzed materials included institutional websites (n = 42), government documents such as bulletins, gazettes, reports, manuals, and national plans (n = 27), legal and regulatory documents (n = 14), scientific articles (n = 12), books (n = 9), press releases (n = 8), and academic theses (n = 5). These materials supported the development of a hierarchical thematic framework based on nodes aligned with the analytical domains of governance, infrastructure, public policy, and equity. The distribution of coded references and documentary sources across thematic nodes is presented in Table 2, with additional details provided in S1 Table.

**Table 2:** Results of thematic coding of AMR-related documents.

Main node	Files^a^	References^b^	Document Types (summary)
Governance analysis	100	288	Government documents, legal regulations, institutional websites
Situational analysis	90	293	Regulations, policy documents, academic articles, theses
Transition toward One Health	33	71	Intersectoral reports, scientific articles, theses
Gender-Based analysis Plus (GBA+)	24	77	Regulations, scientific literature, documents with an equity approach

^a^Files: Total number of documents coded under one or more nodes.

^b^References: Number of document excerpts coded.

To enhance analytical rigor and transparency, representative verbatim quotations from semi-structured interviews were identified for each analytical domain during the thematic coding process. These excerpts were selected as illustrative examples of recurrent patterns and shared perspectives across interviews and are presented as Supporting Information (S3 Table), together with anonymized participant identifiers, institutional sectors, and English translations adapted for academic reporting.

These analytical framework guided the organization of the Results section and supported the identification of cross-cutting barriers, enabling factors, and system-level governance gaps relevant to strengthening AMR governance in Ecuador.

#### Reporting standards.

This study was conducted and reported in accordance with the Standards for Reporting Qualitative Research (SRQR), which guided the transparent reporting of the study design, data collection procedures, analytical process, and interpretation of findings [25]. The completed SRQR checklist is provided as Supporting Information (S1 Checklist).

## Results

The results are presented by analytical domain, integrating findings from document analysis and KI interviews.

### Institutional governance analysis

Triangulation of interview data and document analysis revealed important discrepancies between conceptual awareness and the operational implementation of the OH approach in AMR governance. Findings from interviews with 23 key stakeholders indicated that, although most KIs (96%) reported familiarity with the term “One Health,” only 48% identified the existence of concrete governance structures or institutional mechanisms supporting its implementation. Furthermore, only 30% of participants reported awareness of intersectoral communication tools integrated into institutional practice. Multisectoral collaboration on AMR was also perceived as limited, with 74% of KIs indicating the absence of a clearly defined network of interinstitutional partners or collaborative contacts. Consistent with these perceptions, both interview findings and document analysis suggested that weak institutional articulation among government sectors and agencies responsible for OH implementation constrained the operationalization of the *National Plan for the Prevention and Control of Antimicrobial Resistance (2019–2023)* [10].

The absence of formal coordination mechanisms between ministries and governmental agencies limits the effectiveness of the national AMR response. Although a national AMR committee was officially established to support implementation of the National Plan, both interview findings and document analysis indicated that the committee is currently inactive. This institutional fragmentation –confirmed by the majority of interview participants– appears to be further exacerbated by limited regulatory oversight and weak enforcement mechanisms related to the rational use of antimicrobials across human, animal, and environmental sectors. These findings suggest that the existence of formal policy structures alone is insufficient to ensure effective multisectoral governance and operational integration under the OH approach.

### Situational analysis of AMR governance and infrastructure

One of the major barriers to effective AMR governance identified through document analysis and stakeholder interviews was the fragility of the epidemiological surveillance infrastructure. In particular, policy and technical documents reported that the Integrated Epidemiological Surveillance System (SIVE) [26,27] requires technological modernization and the development of interoperable mechanisms across sectors to improve data integration and coordination. These documents also consistently describe persistent limitations affecting reference laboratories, including insufficient funding, shortages of trained personnel, and inadequate technical equipment, which constrained the routine generation, integration, and exchange of AMR surveillance data.

Several policy documents further indicated that these surveillance and infrastructure limitations hindered the generation of evidence necessary to support timely and evidence-informed decision-making for AMR prevention and control.

### Transitions toward one health: Barriers and facilitating factors

Interview data identified multiple barriers affecting effective collaboration within the OH approach. Key informants frequently described bureaucratic processes, limited political prioritization of AMR, insufficient technical understanding of the OH approach, financial resource constraints, institutional resistance to change, and perceived corruption as major obstacles to intersectoral coordination and collaborative governance. These findings suggest that, beyond formal policy frameworks, structural and institutional factors continue to constrain the operational integration of the OH approach within AMR governance processes.

Triangulation of interview data and document analysis indicated that community misinformation and the inappropriate use of antimicrobials remain persistent challenges, particularly among vulnerable populations and in rural settings. Key informants frequently highlighted self-medication practices, limited public awareness, and barriers to accessing healthcare services as factors contributing to inappropriate antimicrobial use within community contexts. These findings suggest that inequities in healthcare access, together with insufficient regulatory oversight and limited community engagement, continue to undermine AMR prevention efforts at the local level.

Document analysis identified limited and sporadic public awareness initiatives related to antimicrobial use, with few sustained or coordinated communication strategies described in national policy and regulatory documents. Self-medication and unrestricted access to antibiotics - widely documented in the literature [28,29] - were consistently identified as persistent challenges and were perceived to be exacerbated by fragmented and insufficiently coordinated communication and public engagement efforts. Overall, the findings suggest that current strategies addressing antimicrobial use remain weakly integrated across institutional and community levels, thereby limiting the effectiveness of AMR awareness and prevention initiatives.

Interview data further indicated that the main facilitating factors for strengthening the OH approach included improved intersectoral communication, regular meetings and collaborative workshops, technical training, political leadership, and the inclusion of civil society actors in AMR-related initiatives. More than 90% of participants considered stronger intersectoral collaboration to be both achievable and necessary within the Ecuadorian context. Table 3 summarizes key interview findings related to governance, intersectoral coordination, and equity dimensions. These results reflect participants’ perceptions regarding interinstitutional arrangements and operational practices within Ecuador’s AMR governance landscape, where responsibilities are distributed across multiple ministries and agencies with varying levels of institutional coordination and operational integration. Detailed findings are presented in S2 Table.

**Table 3:** Summary of key informant interview findings on governance, equity, and intersectoral coordination related to AMR in Ecuador.

Category	Key response	Percentage (%)
Familiarity with One Health	Yes	95.65
Use of intersectoral communication tools	Yes	30.43
Presence of an AMR contact network	No	73.91
Feasibility of multisectoral collaboration	Yes	91.30
Equity integrated into AMR policies	Yes	4.35
Most frequently cited barrier	Political will	21.74
Most frequently cited facilitator	Meetings/workshops/political engagement	21.74

### Equity analysis using a GBA+ lens

Interview data indicated that participants identified historically underserved populations as priority groups within the context of AMR, including rural communities, low-income populations, migrants, small-scale producers, and immunocompromised individuals. In contrast, document analysis revealed limited and fragmented integration of equity and gender-related considerations within existing AMR policies and regulatory frameworks. When further explored during interviews, some respondents referred specifically to gender equity, others emphasized broader social and economic inequities, and a smaller proportion addressed both dimensions simultaneously. However, fewer than 5% of interviewees considered gender equity or broader social equity to be effectively integrated into current AMR policies. Most respondents (74%) indicated that these dimensions remain either absent or only marginally addressed within existing governance frameworks, while the remaining participants expressed uncertainty or limited knowledge regarding their inclusion.

Triangulation of document analysis and KI interviews confirmed these findings, with Table 4 summarizing the main structural barriers and enabling factors associated with the implementation of the OH approach to address AMR in Ecuador. Six major barriers were identified, including limited intersectoral coordination, insufficient budget allocation, inadequate infrastructure, high staff turnover, limited political prioritization of AMR, and insufficient public education combined with widespread self-medication practices. In contrast, six enabling factors were identified, including the existence of a national AMR surveillance system, a supportive legal and regulatory framework, institutional willingness to collaborate, adoption of international programs and initiatives, the active involvement of academia in policy development, and the adaptation of international guidelines and protocols to the national context.

**Table 4:** Structural barriers and facilitating factors for addressing AMR in Ecuador under a One Health approach.

Barriers	Facilitators
Lack of intersectoral integration and coordination, reflected in the absence of joint working groups across human, animal, and environmental health sectors.	AMR surveillance system: the CRN-RAM (INSPI) provides a foundation for AMR surveillance data generation and potential intersectoral collaboration.
Insufficient budget allocation associated with fiscal constraints, competing national priorities, and public debt.	Supportive legal and regulatory framework: including decrees and resolutions that support AMR-related plans under the OH approach.
Inadequate infrastructure, including underperforming laboratories, budget cuts, and insufficient technical personnel.	Institutional willingness to collaborate among public sector institutions and academic stakeholders.
High staff turnover and limited continuity of technical training programs.	Adoption of international initiatives such as Antimicrobial Stewardship Programs (PROA).
Limited political prioritization of AMR within national government agendas.	Active involvement of academia in generating scientific evidence to inform public policy.
Limited public education and widespread self-medication practices.	Adaptation of international guidelines and protocols to the national context.

## Discussion

This discussion is organized around the four analytical domains guiding the study –governance, infrastructure, public policy, and equity– and integrates findings from document analysis and stakeholder interviews to interpret system-level challenges, implementation gaps, and opportunities for strengthening AMR governance in Ecuador under the One Health (OH) approach. The discussion sections are aligned with the analytical framework presented in the Results and aim to contextualize the findings within broader institutional and multisectoral governance processes.

### Intersectoral governance analysis: challenges under the One Health approach

The absence of formal coordination mechanisms between ministries and governmental agencies limits the effectiveness of the national AMR response. Although the national AMR committee was established to support implementation of the National Plan, available evidence suggests that it is currently inactive or operates with limited operational capacity. This institutional fragmentation appears to be further exacerbated by insufficient regulatory oversight and weak enforcement mechanisms governing the rational use of antimicrobials across the human, animal, and environmental sectors.

Taken together, these findings highlight persistent challenges in achieving effective multisectoral coordination and operational integration under the OH approach, underscoring broader structural weaknesses in AMR governance in Ecuador. The interpretations presented in this section are based on the triangulation of interview data and document analysis, reflecting both documented institutional arrangements and stakeholder perceptions regarding governance performance. While the relevance of the OH approach as a strategic framework for addressing AMR is reinforced by the study findings, its implementation remains constrained by limited political prioritization of AMR, fragmented governance structures and weak interinstitutional coordination, insufficient regulatory oversight of antimicrobial use, and inadequate surveillance capacity for both AMR and antimicrobial consumption.

Similar implementation gaps have been described in other countries adopting OH-based AMR strategies, where the existence of national action plans and formal governance structures has not necessarily translated into effective multisectoral coordination and operational implementation. Previous studies have highlighted that translating OH principles into sustained AMR governance actions requires not only policy alignment, but also functional coordination mechanisms, clearly defined institutional responsibilities, sustainable resources, accountability structures, and continuous cross-sectoral collaborations [12,30]. Therefore, the Ecuadorian experience reflects broader global challenges associated with moving from OH policy formulation toward effective implementation and long-term institutionalization.

### Situational analysis of AMR governance and infrastructure: implementation constraints

These findings highlight persistent structural limitations identified through the triangulation of document analysis and KI interviews, including inadequate infrastructure, insufficient budget allocation, high staff turnover, limited continuity of training programs, insufficient public education, widespread self-medication practices, and the limited political prioritization of AMR within governmental agendas. Table 4 synthesizes six major structural barriers and six enabling factors shaping implementation of the OH approach for addressing AMR in Ecuador. Recognizing these contextual constraints is essential to understanding the feasibility, scope, and sequencing of proposed policy and system-level interventions.

In particular, weaknesses in epidemiological surveillance infrastructure –such as limited interoperability between information systems and the under-resourcing of reference laboratories– have important implications for evidence-informed decision-making. As highlighted in policy and technical reports, these constraints limit the routine generation, integration, exchange, and use of surveillance data necessary to guide AMR prevention and control strategies. Consequently, even where formal policy frameworks exist, their effective implementation remains constrained by insufficient technical capacity, institutional coordination, and operational support systems.

These findings are consistent with evidence from other low- and middle-income settings showing that AMR surveillance systems frequently face challenges related to laboratory capacity, fragmented data systems, limited interoperability, and insufficient sustainable financing, reducing their ability to generate timely evidence for policy decisions [31].

In the broader Latin American context, many countries face structural barriers to AMR control similar to those identified in Ecuador, including limited laboratory capacity, fragmented governance structures, and underfunded public health systems. However, several regional experiences suggest that these challenges can be partially mitigated through sustained institutional coordination and targeted policy interventions. In Argentina, implementation of antimicrobial stewardship programs (PROA) in hospitals –supported by national guidelines, preauthorization protocols, and regular audits– has been associated with reductions in inappropriate antimicrobial use and improvements in antimicrobial prescribing practices [32]. Similarly, Brazil and Colombia have strengthened AMR governance through the establishment of multisectoral coordination committees with formal mandates, designated budget allocations, and integration of the OH approach into national strategies [33]. At the regional level, initiatives such as the Latin American and Caribbean Network for Antimicrobial Resistance Surveillance (ReLAVRA+), coordinated by PAHO, have contributed to improving data standardization, strengthening laboratory capacity, and enhancing cross-border collaboration in AMR surveillance [34].

In contrast, high-income country settings where AMR governance has demonstrated greater effectiveness typically combine legally mandated intersectoral coordination mechanisms, sustained financing for surveillance and laboratory systems, and strong regulatory enforcement across the human, animal, and environmental health sectors [35–37]. The partial, fragmented, or inconsistent presence of these enabling conditions in Ecuador and other low- and middle-income countries may help explain the persistence of implementation gaps despite the formal adoption of national AMR action plans. These findings reinforce the importance of moving beyond policy formulation toward the development of operational governance capacities capable of supporting long-term multisectoral implementation under the OH approach.

Positioning Ecuador’s AMR response within this broader regional and international landscape may help foster an enabling environment for the adaptation and transfer of effective governance and implementation strategies. These may include scaling up hospital-level PROA adapted to local epidemiological contexts, formalizing and sustainably resourcing the National AMR Committee, leveraging regional surveillance and technical cooperation networks, and implementing targeted public education campaigns to address self-medication practices. Collectively, these experiences suggest that structural barriers to AMR governance can be partially mitigated through evidence-informed, context-specific, and operationally feasible interventions aligned with the OH approach.

### Transitions toward One Health: barriers, facilitators, and opportunities for system strengthening

To strengthen the national response to AMR, the establishment of a structured governance framework integrating the human, animal, and environmental health sectors–while also incorporating academic and civil society actors–is essential. This process would require strengthening the National AMR Committee through adequate and sustainable resourcing, a clearly defined mandate, and the active participation of academic institutions and civil society organizations. A consolidated national AMR coordination body could help centralize intersectoral efforts, optimize resource allocation, and promote policy continuity across sectors. Moreover, adaptation of international models–such as antimicrobial stewardship programs (PROA)–could contribute to strengthening national technical and institutional capacity by tailoring protocols, training strategies, and monitoring tools to local epidemiological contexts and resource availability [38,39].

### Equity analysis using GBA+ lens: gender and social considerations

Our findings suggest that limited institutional awareness and operational integration of equity and gender considerations among key stakeholders may contribute to their weak incorporation into AMR-related governance, regulatory, and institutional frameworks. Taken together, the results highlight the need for more systematic integration of gender-responsive and equity-oriented approaches into AMR governance, policy design, and implementation processes under the OH framework. These findings further suggest that insufficient consideration of social vulnerability and inequitable access to healthcare services may limit both the effectiveness and inclusiveness of AMR prevention and response strategies.

Similar challenges have been reported in global AMR governance discussions, where equity, gender, and broader social determinants of health remain insufficiently integrated into national action plans and implementation strategies. Evidence from different settings suggests that socioeconomic inequalities, gender-related factors, geographic barriers, and unequal access to healthcare services can influence antimicrobial access, use, and exposure to AMR risks. Incorporating intersectional approaches, such as the Gender-Based Analysis Plus (GBA+) framework, may strengthen AMR policies by ensuring that interventions address the differentiated needs, vulnerabilities, and capacities of diverse population groups [22,40].

The analysis further revealed limited incorporation of equity-oriented approaches–particularly regarding gender considerations–within AMR-related public policies and institutional frameworks. Vulnerable populations, including rural women, immunocompromised individuals, and people living in conditions of poverty, continue to face significant barriers in accessing appropriate diagnostic and treatment services. These findings reinforce the importance of systematically integrating the Gender-Based Analysis Plus (GBA+) framework into AMR-related regulations, policies, and public health programs to support more inclusive, equitable, and context-responsive interventions under the OH approach.

### A context-specific action framework for strengthening AMR governance in Ecuador under a One Health approach

**Fig 2:**
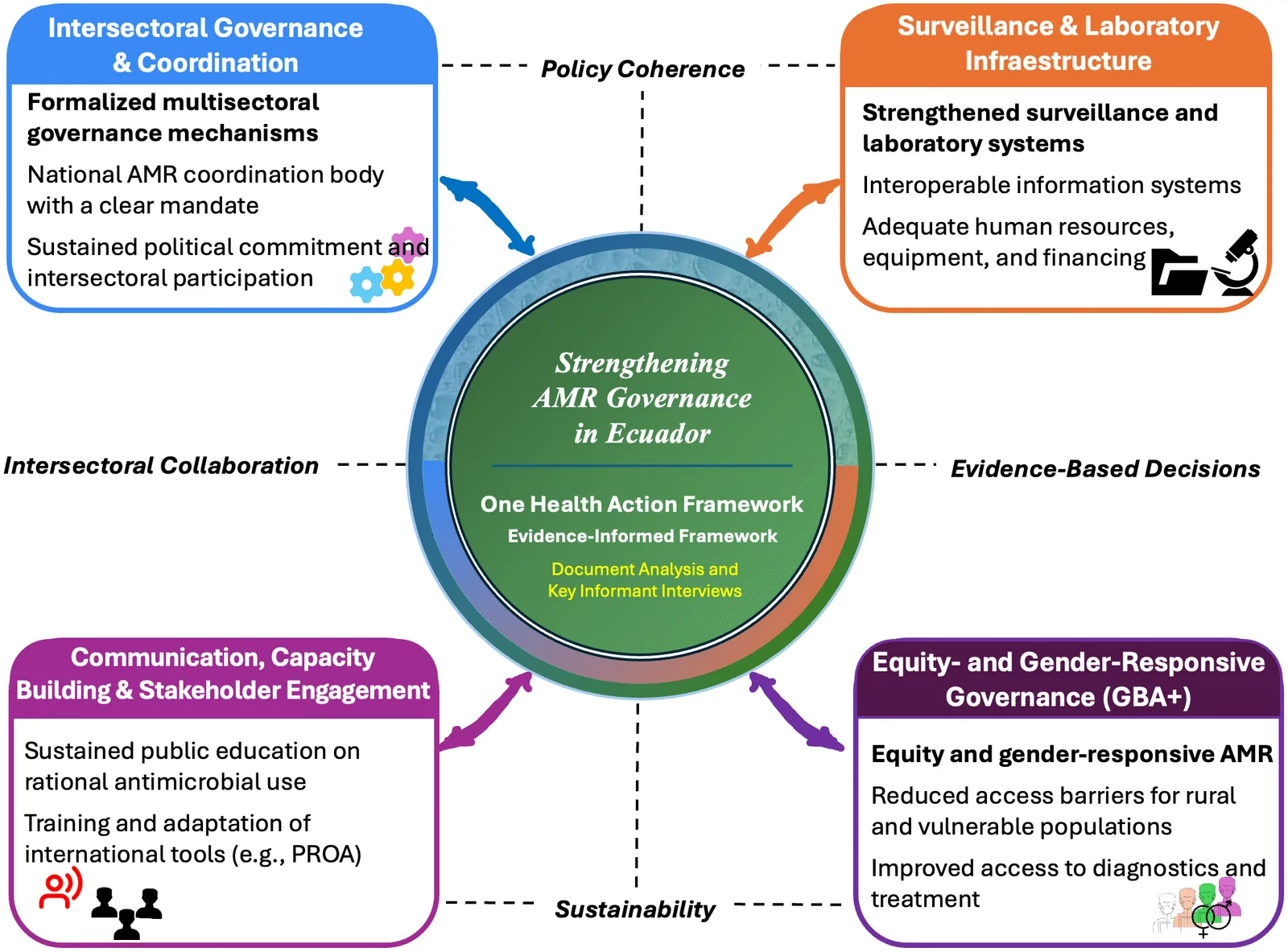
Context-specific action framework for strengthening AMR governance in Ecuador under a One Health approach. **Note.** The framework synthesizes findings from document analysis and KI interviews and is structured around four interrelated pillars: (1) intersectoral governance and coordination; (2) epidemiological surveillance and laboratory infrastructure; (3) equity- and gender-responsive policy integration using a GBA+ lens; and (4) communication, capacity building, and stakeholder engagement. Together, these pillars provide an integrated pathway for strengthening AMR governance in Ecuador and similar low- and middle-income country settings. Open-access icons used in this figure were obtained from SVG Repo (https://www.svgrepo.com/). Attribution and licensing information are as follows: Doctor (SVG Repo, CC0); Gear (thewolfkit, CC BY); Folder Open (Noah Jacobus, Public Domain); Microscope (Icooon Mono, Public Domain); and Speak (Neuicons, MIT License).

Based on the integrated findings from document analysis and KI interviews, this study proposes a context-specific action framework to strengthen AMR governance in Ecuador under the OH approach (Fig 2). The framework is informed by the structural, institutional, operational, and social challenges identified within the Ecuadorian context and is organized around four interrelated and complementary pillars.

These pillars are consistent with international recommendations emphasizing that effective AMR governance requires integrated multisectoral coordination, sustainable surveillance systems, strengthened laboratory capacities, antimicrobial stewardship strategies, and inclusive stakeholder participation across human, animal, and environmental health sectors [36]. Similar approaches have been proposed in other contexts to bridge the gap between national AMR policy development and effective implementation through stronger governance mechanisms and adaptive OH-based strategies [30,41].

The first pillar emphasizes *strengthening intersectoral governance and coordination* through the formalization and sustainable resourcing of a national AMR coordination body with a clearly defined mandate, sustained political commitment, and active participation from the human, animal, and environmental health sectors, as well as academia and civil society actors. The second pillar focuses on *reinforcing epidemiological surveillance and laboratory infrastructure* by improving system interoperability, strengthening technical and human resource capacity, investing in laboratory equipment, and ensuring stable budget allocations to support evidence-informed decision-making and integrated AMR surveillance.

The third pillar addresses the *institutionalization of equity- and gender-responsive policy approaches,* including the systematic integration of a GBA+ lens into AMR policies, regulatory frameworks, and implementation strategies, with particular attention to rural, low-income, and otherwise marginalized populations. The fourth pillar highlights the importance of *strengthening communication, capacity building, and stakeholder engagement* through sustained public education campaigns on rational use of antimicrobials, targeted technical training, and the strategic adaptation of international tools such as PROA to local epidemiological and resource contexts.

Together, these four pillars aim to strengthen coordinated multisectoral governance, reinforce institutional and surveillance capacities, incorporate equity-oriented approaches, and support the long-term implementation and sustainability of AMR response strategies. Collectively, they constitute a pragmatic and transferable framework that moves beyond diagnostic assessment toward actionable guidance, offering a pathway for strengthening AMR governance in Ecuador and other low- and middle-income countries facing similar structural and implementation constraints under the OH approach.

### Limitations

This study has several limitations that should be considered when interpreting the findings. Although the study incorporated perspectives from multiple sectors involved in AMR governance, representation across OH domains was not fully balanced. In particular, the environmental health sector was represented by a limited number of participants compared with the human and animal health sectors. This imbalance may also reflect broader structural challenges affecting the environmental sector in Ecuador, including limited technical capacity, reduced institutional participation in AMR-related initiatives, competing political and administrative priorities, and broader socioeconomic constraints affecting intersectoral engagement within the OH framework. Additionally, as a qualitative governance study, the findings are interpretative, context-specific, and intended to support analytical transferability rather than statistical generalization.

## Conclusions

Ecuador faces structural, institutional, and social challenges that constrain its national response to antimicrobial resistance (AMR). Based on the triangulation of document analysis and key informant interviews, this study identified that despite the existence of a national policy framework, fragmented governance, limited operational coordination, weaknesses in surveillance infrastructure, insufficient regulatory enforcement, and limited integration of equity considerations continue to hinder effective AMR implementation under the One Health (OH) approach. Addressing these challenges requires sustained political commitment, strengthened multisectoral coordination mechanisms, improved institutional and technical capacities, and the systematic incorporation of equity-oriented perspectives into AMR policies and programs. The context-specific action framework proposed in this study provides evidence-informed guidance to support more integrated, sustainable, and inclusive AMR governance in Ecuador and may also offer relevant insights for other low- and middle-income countries facing similar AMR governance and implementation challenges.

## Supporting information

S1 Table Results of thematic coding of AMR-related documents. **Description:** Detailed results of the NVivo thematic coding of documents included in the environmental scan, organized by analytical domain, sub-themes, document types, and synthesized key findings.
(XLSX)

S2 Table Results of key informant interviews on governance, equity, and intersectoral barriers. **Description:** Expanded summary of findings from semi-structured interviews with key informants, presented by analytical domain and reporting the distribution of responses related to governance, intersectoral coordination, and equity considerations.
(DOCX)

S3 Table Representative verbatim quotes by analytical domain (Spanish and English). **Description:** Selection of anonymized verbatim quotes from semi-structured interviews, organized by analytical domain and institutional sectors, including the original Spanish excerpts and their academic English translations.
(DOCX)

S1 Checklist Standards for Reporting Qualitative Research (SRQR) checklist. **Description:** Complete Standards for Reporting Qualitative Research (SRQR) checklist indicating where each reporting criterion is addressed in the manuscript.
(DOCX)

S1 Text Detailed NVivo coding framework and node structure. **Description:** Documentation of the NVivo coding framework used in this study, including the hierarchical structure of nodes and sub-nodes applied to documents analysis and interview transcripts.
(DOCX)
