# Supplementary material for: Antimicrobial resistance in Ecuador: A One Health situational analysis of governance, infrastructure, and equity gaps
Source: PLOS Glob Public Health. 2026 Aug 21;6(8):e0005308. doi: 10.1371/journal.pgph.0005308 (PMC13496403; doi:10.1371/journal.pgph.0005308)
Supplement: Supplementary file 1 [file pgph.0005308.s008.pdf]

Response to Reviewers

Manuscript ID:	PGPH-D-25-02802R1
Title:	Antimicrobial resistance in Ecuador: A One Health situational analysis of governance, infrastructure, and equity gaps
Journal:	PLOS Global Public Health

Dear Dr. Sarah Jose

We sincerely thank you and the reviewers for the careful evaluation of our manuscript and for the constructive and insightful comments provided throughout the review process. We appreciate the opportunity to revise and resubmit our work to PLOS Global Public Health.

In response to the reviewers' comments, we have thoroughly revised the manuscript to strengthen its analytical depth and interpretation of findings, particularly in the Results and Discussion sections. We clarified the qualitative scope and governance-oriented objectives of the study, strengthened the articulation and coherence of the One Health (OH) framework throughout the manuscript and expanded the discussion regarding implementation challenges, intersectoral coordination, surveillance infrastructure, and equity considerations related to antimicrobial resistance (AMR) in Ecuador.

In addition, we carefully revised the manuscript for scientific English, coherence, terminology consistency, and methodological clarity. We also refined the structure of the analytical domains, strengthened the interpretation of qualitative findings through improved triangulation between document analysis and stakeholder interviews, revised and standardized references, and incorporated dedicated limitations subsection and an expanded data availability statement in accordance with the reviewers' recommendations.

Below, we provide a detailed point-by-point response to each reviewer comment and indicate the corresponding revisions made in the manuscript.

We hope that the revised version adequately addresses all concerns raised during the review process and that the manuscript will now be suitable for publication in PLOS Global Public Health.

Reviewer # 1 – General comments

Comment:

"The manuscript addresses an important and timely public health issue, and the authors should be commended for the substantial improvements made in this revised version, particularly in clarifying the qualitative design and strengthening the overall structure of the paper. The study has clear potential to make a valuable contribution to the literature on AMR governance and One Health in Ecuador and the region. However, a small number of remaining editorial, consistency, and clarity issues should still be addressed before the manuscript is ready for publication."

Response:

We sincerely thank Reviewer #1 for the careful evaluation of our manuscript and for the constructive and encouraging comments. We appreciate the recognition of the

improvements made in the revised version, particularly regarding clarifications of the qualitative design and strengthening of the manuscript structure. Following the reviewer's recommendations, we carefully revised the manuscript to address the remaining editorial, consistency, clarity, and language-related issues. Detailed responses to each comment are provided below.

Revision 1

Reviewer comments: *"Please, do replace all placeholder text in the figure captions and notes. Figure 1 still contains placeholder text ("This is the Fig1 Title" and "This is the Fig 1 Note"), and Figure 2 also still contains placeholder text in both the title and note. These must be replaced with the final, publication ready caption text."*

Response

We thank the reviewer for identifying these remaining placeholder texts. Figure captions and notes for Figures 1 and 2 were carefully revised and replaced with final publication-ready titles and explanatory notes consistent with the manuscript content.

Revision 2

Reviewer comments: *"Please, do correct the typo in Figure 2 (ARM should be AMR) and check figure numbering consistency. The Figure 2 caption currently reads "strengthening ARM governance," which is clearly incorrect. It also refers to "This is the Fig 1 Title," suggesting that figure labeling was not finalized."*

Response

We appreciate the reviewer for identifying these inconsistencies. The typo in Figure 2 ("ARM" corrected to "AMR") was corrected, and all figure titles, captions, numbering, and notes were carefully reviewed and standardized throughout the manuscript.

Revision 3

Reviewer comments: *"The manuscript still requires a final, careful language and copyediting revision, since there are residual grammatical and wording problems throughout the text, for example: "a preventive measures," "have hindering," "Search terms included," "AMR polices," "low income," "country setting," and "subjected to ethical approval." A thorough proofread is still needed."*

Response

We thank the reviewer for highlighting the remaining language and copyediting issues. The manuscript underwent an additional careful revision focused on scientific English, grammar, terminology consistency, spelling, and overall readability. The specific examples identified by the reviewer were corrected, and additional editorial revisions were implemented throughout the manuscript.

Revision 4

Reviewer comments: *"Please, do correct remaining terminology and spelling issues in Table 1. For example, "Enviromental health" should be "Environmental health," and*

"affiliates agencies" should be revised for grammatical correctness (e.g., "affiliated agencies")."

Response

We appreciate the reviewer's observation. The terminology and spelling inconsistencies identified in Table 1 were corrected, including revision of *"Enviromental health" to "Environmental health"* and grammatical standardization of terms such as "affiliated agencies." The table was further reviewed for consistency and clarity.

Revision 5

Reviewer comments: *"Please, do further clarify the evidentiary basis of the macro political and austerity related claims. The Introduction still includes strong statements about the shift to a neoliberal model, austerity measures, budget cuts, and institutional weakening. These points may be valid, but they should be signposted more explicitly as either: (a) contextual background from secondary literature, (b) document-based findings, or (c) stakeholder-reported perceptions."*

Response

We thank the reviewer for this important recommendation. The manuscript was revised to clarify the evidentiary basis of statements related to austerity policies, institutional restructuring, and governance challenges. These statements are now more explicitly contextualized as interpretations derived from secondary literature, document analysis, or stakeholder-reported perceptions, as appropriate. Additional clarifying language was incorporated in the Introduction and Discussion sections to improve transparency regarding the sources and interpretation of these contextual claims.

Revision 6

Reviewer comments: *"Please, do make the basis for budgetary and resource related claims more explicit at the point of use. The manuscript refers to insufficient budget allocation, financial constraints, and limited human and material resources. Where these are based on documentary evidence, the relevant sources should be made explicit, where they are based mainly on stakeholder perceptions, the wording should be softened accordingly."*

Response

We appreciate the reviewer's recommendation. References to budgetary, financial, technical, and human resource constraints were revised throughout the manuscript to clarify whether they were derived from documentary evidence, stakeholder perceptions, or triangulation between both sources. Wording was refined where appropriate to improve analytical precision and methodological transparency.

Revision 7

Reviewer comments: *"Please, you should add a brief limitations paragraph that explicitly acknowledges sectoral imbalance in the interview sample. This would strengthen the methodological transparency of the paper. In particular, environmental health appears to be represented by only one participant, which should be acknowledged as a limitation when discussing transferability across all One Health sectors."*

Response

We thank the reviewer for this valuable methodological observation. A dedicated "Limitations" subsection was incorporated into the Discussion section acknowledging the imbalance in sectoral representation within the interview sample, particularly the more limited participation from the environmental health sector. The revised text also discusses how this imbalance may reflect broader structural and institutional challenges affecting environmental sector participation in AMR governance processes within the Ecuadorian context.

Final Comment

Reviewer comments: *"Although the manuscript has improved substantially and I have identified few of the remaining issues that still need correction, I encourage the authors to undertake a careful and meticulous final revision of the entire manuscript. This is a valuable and timely study with clear potential to contribute meaningfully to the literature on AMR governance and One Health in Ecuador and the region. With these final corrections, the paper will be in a much stronger position for publication."*

Response

We sincerely appreciate the reviewer's positive assessment of the manuscript and the constructive recommendations provided throughout the review process. We carefully implemented the suggested revisions and conducted an additional detailed review of the manuscript to improve clarity, consistency, methodological transparency, and scientific English. We hope that the revised version adequately addresses all remaining concerns.

Reviewer # 2 – General comments

Comment:

"Although the topic and findings are relevant for the country and the broader region, the manuscript still needs stronger analysis to support its conclusions. The organization and writing have improved, especially in how the results are presented, but the depth of analysis is still limited. The discussion, in particular, lacks critical interpretation of the findings.

In addition, several references (at least 10 out of 33) could not be accessed because the cited websites are no longer available. These seem to be important sources supporting the funding claims made by the authors. Without access to these documents, it is difficult to assess or verify how that information is being interpreted."

Response

We sincerely thank the reviewer for the thoughtful and constructive evaluation of our manuscript. We particularly appreciate the reviewer's recognition of the relevance of the topic and the improvements made in the organization and presentation of the manuscript. In response to the reviewer's concerns, we substantially revised the manuscript to

strengthen the analytical depth, interpretation, and discussion of the findings, particularly regarding AMR governance, implementation challenges, intersectoral coordination, surveillance infrastructure, and equity considerations under the One Health (OH) approach.

More specifically, the Results and Discussion sections were extensively revised to improve the triangulation and interpretation of findings derived from document analysis and key informant interviews. We expanded the analytical framing of governance and implementation barriers, clarified the evidentiary basis of contextual and institutional claims, strengthened the interpretation of structural and operational constraints affecting AMR response implementation, and incorporated broader regional and international comparisons to contextualize the Ecuadorian case. In addition, we refined the conceptual integration of equity-oriented and gender-responsive approaches within AMR governance and further developed the context-specific action framework proposed in the Discussion section.

We also carefully revised the manuscript for scientific English, coherence, terminology consistency, and methodological clarity. Finally, the reference list and all cited URLs were reviewed and updated whenever possible to improve accessibility, consistency, and transparency of supporting sources.

Finally, we thank the reviewer for highlighting the accessibility issues affecting several cited references. In response, all references and URLs were carefully reviewed and updated whenever alternative or active sources were available. In addition, the manuscript text was revised to improve transparency regarding the evidentiary basis of statements related to governance, funding constraints, institutional capacity, and policy implementation. Where appropriate, contextual claims were more explicitly framed as interpretations derived from secondary literature, document analysis, or stakeholder-reported perceptions.

Final Comments

We sincerely thank the Academic Editor and the reviewers for the time, expertise, and constructive feedback dedicated to the evaluation of our manuscript. We appreciate the positive assessment of the relevance of the study and the opportunity to further improve the manuscript through this revision process.

The revised manuscript was carefully refined in response to the reviewers' comments, with particular attention to improving conceptual clarity, consistency in the interpretation of findings, methodological transparency, organization of the analytical sections, and overall scientific language. Revisions focused mainly on clarifying the qualitative and governance-oriented scope of the study, strengthening the articulation and coherence of the One Health (OH) framework throughout the manuscript, improving the interpretation and contextualization of findings derived from document analysis and key informant interviews, and refining the Results and Discussion sections according to the analytical domains of the study. Additional revisions included correction of terminology and formatting inconsistencies, careful language and copyediting revision, clarification of the evidentiary basis of contextual and governance-related claims, updating and verification of references and URLs, incorporation of a dedicated limitations subsection, and refinement of the Data Availability Statement and supporting materials.

We hope that the revised manuscript adequately addresses all concerns raised during the review process and meets the publication standards of PLOS Global Public Health. We are grateful for the opportunity to revise and resubmit our work and would be pleased to provide any additional clarifications if required.

Kind regards,

Validar únicamente en FirmaEC.
Firmado electrónicamente por:
RICHAR IVAN
RODRIGUEZ HIDALGO

Richar Rodríguez-Hidalgo, PhD

On behalf of all authors
