# Supplementary material for: Antimicrobial resistance in Ecuador: A One Health situational analysis of governance, infrastructure, and equity gaps
Source: PLOS Glob Public Health. 2026 Aug 21;6(8):e0005308. doi: 10.1371/journal.pgph.0005308 (PMC13496403; doi:10.1371/journal.pgph.0005308)
Supplement: Supplementary file 2 [file pgph.0005308.s009.pdf]

Response to Reviewers

Manuscript ID:	PGPH-D-25-02802R2
Title:	Antimicrobial resistance in Ecuador: A One Health situational analysis of governance, infrastructure, and equity gaps
Journal:	PLOS Global Public Health

Dear Dr. Bipin Adhikari

Academic Editor

We sincerely thank you for the careful evaluation of our manuscript and for your constructive and insightful comments provided during this final stage of the review process. We appreciate your positive assessment of the relevance of our study within current One Health (OH) and antimicrobial resistance (AMR) discourse, as well as the opportunity to further strengthen the manuscript.

In response to your recommendations, we have carefully revised the manuscript to strengthen its conceptual foundation, methodological transparency, and interpretation of findings. Specially, we more explicitly defined the OH framework guiding the study and clarified the theoretical and governance-oriented analytical approach used to examine AMR governance in Ecuador. We also expanded the description of the analytical framework by explaining the selection of stakeholder-related dimensions, including institutional roles, responsibilities, priorities, interactions, and levels of involvement across sectors.

Additional revisions included clarifying the rationale for the selection of Canada, Brazil, Rwanda, and Ecuador within the broader comparative study; incorporating supporting references for the Gender-Based Analysis-Plus (GBA+) approach; and strengthening the Discussion section by engaging more extensively with relevant literature on OH implantation, AMR governance, surveillance capacity, antimicrobial stewardship, and equity considerations. These changes aimed to better contextualize how the findings from Ecuador align with broader regional and international experiences.

We also revised the manuscript for clarity, consistency, and scientific language, updated references were appropriate and condensed the Conclusion section into a single integrative paragraph to reduce repetitions and provide a clearer synthesis of the study findings and implications.

Below, we provide a detailed point-by-point response to each your comments and indicate the corresponding revisions made in the manuscript.

We hope that the revised version adequately addresses your recommendations and further strengthens the contribution of the study to PLOS Global Public Health.

Academic Editor comments

Suggestion 1:

Academic Editor comment *"In the Methods section, clearly state or reference the One Health framework. Alternatively, add a line on One Health framework and cite a reference."*

Response:

We thank the Academic Editor for this valuable suggestion. We revised the Methods section to explicitly define the OH framework used to guide the study. Specifically, we incorporate a statement describing the OH approach as an integrated governance framework emphasizing multisectoral coordination, the interdependence between human, animal, and environmental health system, and collaborative institutional response to complex public health challenges such as AMR. An appropriate reference supporting the OH framework was also added.

Revision made:

The following text was incorporated into the Materials and Methods section:

"*The study was conceptually informed by the OH approach as an integrated governance framework emphasizing multisectoral coordination, interdependence between human, animal, and environmental health systems, and the need for collaborative institutional responses to complex public health challenges such as AMR [12].*"

Suggestion 2:

Academic Editor comment: *"Line 127: The study design was intended to examine…… Did you have any existing theoretical framework guiding your analysis? There are theoretical frameworks applied to One Health in AMR you might want to build on: https://gh.bmj.com/content/10/10/e019236"*

Response

We thank the Academic Editor for this important observation. We agree that the theoretical and analytical foundation guiding the study needed to be more explicitly described. In response, we revised the Analytical framework and data analysis section to clarify that the study was guided by a OH governance-oriented analytical framework focused on intersectoral coordination, institutional interactions, surveillance capacities, implementation processes, and equity dimensions relevant to AMR governance.

We also incorporated additional literature on OH governance and implementation frameworks to better position our analytical approach within existing theoretical perspectives. These revisions clarify how the OH framework informed the examination of governance structures, stakeholder interactions, operational barriers, and multisectoral implementation challenges affecting AMR response in Ecuador.

Revision made:

The following text was incorporated into Analytical framework and data analysis section:

"*The analytical framework was developed to operationalize the OH approach within a qualitative governance-oriented analysis, emphasizing the interdependence of human, animal, and environmental health systems, as well as the importance of intersectoral coordination, institutional interactions, surveillance capacities, implementation processes, and equity dimensions relevant to AMR governance [12]. This framework guided the examination of governance structures, stakeholder´s interactions, operational*

barriers, and multisectoral implementation challenges affecting AMR response in Ecuador, drawing on contemporary OH governance approaches described in the literature [12,19–23]."

Suggestion 3:

Academic Editor comment: *"Add a line on why you chose these specific countries: Canada, Brazil, Rwanda, and Ecuador."*

Response

We thank the Academic Editor for this helpful suggestion. We revised the Study Desing and Setting section to clarify the rationale for including Canada, Brazil, Rwanda, and Ecuador within the broader comparative study. We specified that these countries were selected to represent diverse geographical, institutional, and socioeconomic contexts, allowing the examinations of AMR governance and implementation challenges across different health systems and policy environments under the OH approach.

Revision made:

The following text was incorporated into the Study design and setting section:

"*These countries were selected to represent diverse geographical, institutional, and socioeconomic contexts for examining governance and implementation challenges related to antimicrobial resistance under the OH approach. The broader study aimed to identify barriers, gaps, and opportunities for strengthening AMR governance across different multisectoral and policy environments.*"

Suggestion 4:

Academic Editor comment: "*Analytical framework and data analysis: Your second thematic analysis clearly presents some elements of stakeholder analysis. Within this section or elsewhere in the methods, clearly mention how you picked these elements (stakeholder roles, institutional positions, priorities…), among others."*

Response

We thank the Academic Editor for this insightful observation. We agree that the rationale for selecting the stakeholder-related dimension required further clarification. The Analytical framework and data analysis section was revised to explicitly describe that these dimensions were selected based on the study objectives and contemporary OH approaches, which emphasize institutional coordination, multisectoral participation, governance interactions, and implementation processes relevant to AMR response.

We clarified that stakeholder roles, institutional responsibilities, priorities, levels of involvements, intersectoral communication mechanisms, and operational capacities were examined as key dimensions to understand how different actors contributed to, interact within, or experience barriers in AMR governance processes across the human, animal, and environmental health sectors.

Revision made:

The following text was incorporated into the Analytical framework and data analysis section:

"*Selection of the analytical domains and stakeholder-related dimensions was informed by the study objectives and by contemporary OH governance approaches emphasizing institutional coordination, multisectoral participation, implementation processes, and governance interactions relevant to AMR response. Particular attention was given to stakeholder roles, institutional responsibilities, levels of involvement, intersectoral communication mechanisms, and operational capacities across the human, animal, and environmental health sectors.*"

Suggestion 5:

Academic Editor comment: "*Equity analysis using a gender-based analysis — have you got a reference to back this analysis?*"

Response

We thank the Academic Editor for this important methodological suggestion. We agree that the theoretical basis supporting the use of the Gender-Based Analysis Plus (GBA+) approach needed to be more explicitly referenced and described. In response, we revised the Analytical framework and data analysis section by incorporating appropriate references supporting the GBA+ framework and clarified how this approach was used as an analytical lens within the study.

Specifically, we clarified that GBA+ was applied to examine how gender and other intersecting social determinants may influence vulnerability, access to services, participation, and equity-related dimensions within AMR governance and policy implementation processes.

Revision made:

The following text was incorporated into the Analytical framework and data analysis section:

"*Equity analysis using a Gender-Based Analysis Plus (GBA+) lens: evaluation of how AMR-related policies and governance processes address gender equity, access to services, and impacts on vulnerable populations. The GBA+ framework was used as an analytical tool to examine how gender and other intersecting social determinants may influence exposure, access to services, participation, and vulnerability within AMR-related governance and policy processes [22,23].*"

Suggestion 6:

Academic Editor comment: "*A lot of paragraphs within the discussion section could be engaged with more extensive literature from your own document analysis. Consider discussing the literature, whether and how your findings differ or echo with others.*"

Response

We thank the Academic Editor for this valuable recommendation. We agree that further engagement with the existing literature was necessary to strengthen the interpretation and contextualization of our findings. In response, we revised the Discussion section to better integrate evidence from the literature and to compare the Ecuadorian findings with experiences reported in other national, regional, and international contexts.

Specifically, we expanded the discussion of AMR governance challenges, One Health implementation gaps, surveillance infrastructure limitations, antimicrobial stewardship strategies, and multisectoral coordination mechanisms. These revisions highlight areas where the Ecuadorian experience aligns with challenges reported in other low- and middle-income countries, while also identifying context-specific institutional and operational barriers affecting AMR governance implementation.

Revision made:

Additional literature and comparative analysis were incorporated throughout the Discussion section. For example, the following text was added:

"*Similar implementation gaps have been described in other countries adopting OH-based AMR strategies, where the existence of national action plans and formal governance structures has not necessarily translated into effective multisectoral coordination and operational implementation. Previous studies have highlighted that translating OH principles into sustained AMR governance actions requires not only policy alignment, but also functional coordination mechanisms, clearly defined institutional responsibilities, sustainable resources, accountability structures, and continuous cross-sectoral collaboration [12,30]. Therefore, the Ecuadorian experience reflects broader global challenges associated with moving from OH policy formulation toward effective implementation and long-term institutionalization*."

Suggestion 7:

Academic Editor comment: "*Line 42: While the relevance… This is a valid point. Again, relate/discuss this with similar literature*."

Response

We thank the Academic Editor for this helpful recommendation. We revised this section of the Discussion to better connect our findings with existing literature on One Health implementation and AMR governance. Specifically, we expanded the interpretation of the gap between the formal recognition of the OH approach and its operational implementation, highlighting that similar challenges have been reported in other contexts where national AMR strategies and governance structures exist but have not always translated into effective multisectoral coordination and sustained implementation.

The revised text further emphasizes that successful OH implementation requires not only policy adoption, but also functional coordination mechanisms, institutional accountability, sustainable resources, and continuous engagement across sectors.

Revision made:

The following text was incorporated into the Discussion section:

"*Similar implementation gaps have been described in other countries adopting OH-based AMR strategies, where the existence of national action plans and formal governance structures has not necessarily translated into effective multisectoral coordination and operational implementation. Previous studies have highlighted that translating OH principles into sustained AMR governance actions requires not only policy alignment, but also functional coordination mechanisms, clearly defined institutional responsibilities, sustainable resources, accountability structures, and continuous cross-sectoral collaboration [12,30]. Therefore, the Ecuadorian experience reflects broader global challenges associated with moving from OH policy formulation toward effective implementation and long-term institutionalization.*"

Suggestion 8:

Academic Editor comment: "*Line 505: Our findings suggest that limited… This is again an important point; discuss this with the literature, whether this is consistent across other geographical regions.*"

Response

We thank the Academic Editor for highlighting this important point. We agree that the findings related to the limited integration of equity and gender considerations into AMR governance required stronger contextualization within the existing literature. In response, we expanded the Equity analysis section of the Discussion to compare our findings with broader evidence from other geographical and institutional contexts.

The revised discussion emphasizes that challenges related to the insufficient incorporation of equity, gender perspectives, and broader social determinants of health into AMR policies are not unique to Ecuador, but have also been reported in global AMR governance discussions. We further clarified the relevance of intersectional approaches, such as the Gender-Based Analysis Plus (GBA+) framework, to support more inclusive and context-responsive AMR policies.

Revision made:

The following text was incorporated into the Equity analysis using GBA+ lens section of the Discussion:

"*Similar challenges have been reported in global AMR governance discussions, where equity, gender, and broader social determinants of health remain insufficiently integrated into national action plans and implementation strategies. Evidence from different settings suggests that socioeconomic inequalities, gender-related factors, geographic barriers, and unequal access to healthcare services can influence antimicrobial access, use, and exposure to AMR risks. Incorporating intersectional approaches, such as the Gender-Based Analysis Plus (GBA+) framework, may therefore strengthen AMR policies by ensuring that interventions address the differentiated needs, vulnerabilities, and capacities of diverse population groups [22,40].*"

Suggestion 9:

Academic Editor comment: "*And then the following paragraphs as well; consider engaging with more literature.*"

Response

We thank the Academic Editor for this recommendation. In response, we further revised the Discussion section to strengthen the connection between our findings, the proposed action framework, and existing literature on AMR governance and One Health implementation. Additional references and contextual discussion were incorporated to support the proposed governance, surveillance, equity, and stakeholder engagement strategies.

Specifically, we clarified that the four pillars proposed in the context-specific action framework are consistent with international recommendations emphasizing multisectoral coordination, sustainable surveillance systems, strengthened laboratory capacity, antimicrobial stewardship strategies, and inclusive stakeholder participation. These revisions better position the proposed framework within broader evidence-based approaches for translating AMR policies into effective One Health implementation.

Revision made:

The following text was incorporated into the section "A context-specific action framework for strengthening AMR governance in Ecuador under a One Health approach":

"*These pillars are consistent with international recommendations emphasizing that effective AMR governance requires integrated multisectoral coordination, sustainable surveillance systems, strengthened laboratory capacities, antimicrobial stewardship strategies, and inclusive stakeholder participation across human, animal, and environmental health sectors [36]. Similar approaches have been proposed in other contexts to bridge the gap between national AMR policy development and effective implementation through stronger governance mechanisms and adaptive OH-based strategies [30,41].*"

Suggestion 10:

Academic Editor comment: "*Reduce your conclusion section to a paragraph (you could turn it into sets of recommendations), as it repeats much of what you have already reported in previous sections.*"

Response

We thank the Academic Editor for this valuable recommendation. We agree that the Conclusions section could be more concise and focused on the main implications of the study. In response, we substantially revised and condensed the Conclusions into a single integrative paragraph to reduce repetition with the Results and Discussion sections.

The revised conclusion now provides a clearer synthesis of the main governance challenges identified, emphasizes the need for strengthened One Health implementation, and highlights the relevance of the proposed context-specific action framework as

evidence-informed guidance for Ecuador and other low- and middle-income countries facing similar AMR governance and implementation challenges.

Revision made:

The Conclusions section was rewritten and condensed into the following paragraph:

"*Ecuador faces structural, institutional, and social challenges that constrain its national response to antimicrobial resistance (AMR). Based on the triangulation of document analysis and key informant interviews, this study identified that despite the existence of a national policy framework, fragmented governance, limited operational coordination, weaknesses in surveillance infrastructure, insufficient regulatory enforcement, and limited integration of equity considerations continue to hinder effective AMR implementation under the One Health (OH) approach. Addressing these challenges requires sustained political commitment, strengthened multisectoral coordination mechanisms, improved institutional and technical capacities, and the systematic incorporation of equity-oriented perspectives into AMR policies and programs. The context-specific action framework proposed in this study provides evidence-informed guidance to support more integrated, sustainable, and inclusive AMR governance in Ecuador and may also offer relevant insights for other low- and middle-income countries facing similar AMR governance and implementation challenges.*"

Final Comments

We sincerely thank the Academic Editor for the time, expertise, and constructive feedback dedicated to the evaluation of our manuscript. We appreciate the positive assessment of the relevance of this study within the current One Health (OH) and antimicrobial resistance (AMR) discourse, as well as the opportunity to further strengthen the manuscript through this revision process.

The revised manuscript was carefully refined in response to the Academic Editor's recommendations, with particular attention to strengthening the conceptual foundation, methodological transparency, interpretation of findings, and integration with existing literature. Revisions focused mainly on more explicitly defining the OH framework guiding the study, clarifying the qualitative and governance-oriented analytical approach, and strengthening the description of stakeholder-related dimensions, institutional interactions, and multisectoral implementation challenges relevant to AMR governance.

Additional revisions included clarification of the rationale for the broader multi-country study design, incorporation of supporting references for the Gender-Based Analysis Plus (GBA+) approach, and expansion of the Discussion section to better contextualize the Ecuadorian findings within regional and international evidence on OH implementation, AMR governance, surveillance capacity, antimicrobial stewardship, and equity considerations. The Conclusions section was also condensed into a single integrative paragraph to reduce repetition and provide a clearer synthesis of the study findings and implications.

We hope that the revised manuscript adequately addresses all recommendations provided during this final evaluation stage and further strengthens the contribution of this study to

PLOS Global Public Health. We are grateful for the opportunity to revise and resubmit our work and would be pleased to provide any additional clarifications if required.

Kind regards,

Validar únicamente en FirmaEC.
Firmado electrónicamente por:
RICHAR IVAN
RODRIGUEZ HIDALGO

Richar Rodríguez-Hidalgo, PhD

On behalf of all authors
