# Supplementary material for: Antimicrobial resistance in Ecuador: A One Health situational analysis of governance, infrastructure, and equity gaps
Source: PLOS Glob Public Health. 2026 Aug 21;6(8):e0005308. doi: 10.1371/journal.pgph.0005308 (PMC13496403; doi:10.1371/journal.pgph.0005308)
Supplement: Supplementary file 3 [file pgph.0005308.s002.docx]

**Results of key informant interviews on governance, equity, and intersectoral barriers**

| **Topic Area** | **Question** | **Response** | **n** | **%** |
| --- | --- | --- | --- | --- |
| General One Health | Q1. Are you familiar with the term 'One Health'? | Yes | 22 | 95.65 |
|  |  | No | 1 | 4.35 |
|  | Q2. Do multisectoral collaboration and intersectoral approaches like One Health contribute to how your institution addresses AMR? | Yes | 21 | 91.30 |
|  |  | No | 2 | 8.70 |
| One Health Governance Landscape | Q3. Do you know of any specific governance infrastructure related to One Health in your country? | Yes | 11 | 47.83 |
|  |  | No | 12 | 52.17 |
|  | Q4. Are you aware of any intersectoral communication tools used in the context of your work? | Yes | 7 | 30.43 |
|  |  | No | 16 | 69.57 |
| Existing One Health Collaborations | Q5. Are there people you can contact to collaborate in One Health? | Yes | 6 | 26.09 |
|  |  | No | 17 | 73.91 |
|  | Q6. Which institutions outside of yours do you interact with regularly to address AMR? | AGROCALIDAD | 6 | 26.09 |
|  |  | Ministry of Health and agencies | 5 | 21.74 |
|  |  | Universities | 2 | 8.70 |
|  |  | International organizations | 2 | 8.70 |
|  |  | Ecuadorian organizations | 3 | 13.04 |
|  |  | None | 5 | 21.74 |
|  | Q7. How involved are non-government stakeholders in One Health for AMR? | High | 1 | 4.35 |
|  |  | Medium | 3 | 13.04 |
|  |  | Low | 10 | 43.48 |
|  |  | None | 6 | 26.09 |
|  |  | Don’t know | 3 | 13.04 |
|  | Q8. Do you have an example of a successful interdisciplinary or multisectoral One Health collaboration on AMR? | Yes | 9 | 39.13 |
|  |  | No | 14 | 60.87 |
|  | Q9. Do you have an example of a failed multisectoral collaboration? | Yes | 7 | 30.43 |
|  |  | No | 16 | 69.57 |
|  | Q10. Are there agencies you should interact with more to address AMR? | State institutions | 11 | 47.83 |
|  |  | Universities | 3 | 13.04 |
|  |  | International organizations | 3 | 13.04 |
|  |  | Don’t know | 6 | 26.09 |
|  | Q11. Do you believe that fostering stronger multisectoral collaboration is feasible or useful? | Yes | 21 | 91.30 |
|  |  | No | 2 | 8.70 |
| Health Equity Considerations | Q12. Which equity-deserving groups are identified in your institutional response to AMR? | Rural areas | 3 | 13.04 |
|  |  | All groups | 7 | 30.43 |
|  |  | Low-income populations | 4 | 17.39 |
|  |  | Small producers | 2 | 8.70 |
|  |  | Children, elderly, impoverished areas | 2 | 8.70 |
|  |  | Migrants | 1 | 4.35 |
|  |  | Immunocompromised | 1 | 4.35 |
|  |  | Don’t know | 3 | 13.04 |
|  | Q13. Do you believe health equity is relevant to AMR? | Yes | 11 | 47.83 |
|  |  | No | 10 | 43.48 |
|  |  | No response | 2 | 8.70 |
|  | Q14. Are equity considerations integrated into One Health programs and policies for AMR? | Yes | 1 | 4.35 |
|  |  | No | 17 | 73.91 |
|  |  | No response | 5 | 21.74 |
| Barriers and Facilitators | Q15. What are the main barriers to intersectoral collaboration? | Political will | 5 | 21.74 |
|  |  | Bureaucracy | 4 | 17.39 |
|  |  | Knowledge gaps | 3 | 13.04 |
|  |  | Economic resources | 2 | 8.70 |
|  |  | Vanity/Egos | 2 | 8.70 |
|  |  | Resistance to change | 1 | 4.35 |
|  |  | Ethics | 1 | 4.35 |
|  |  | Corruption | 1 | 4.35 |
|  |  | Noncompliance with regulations | 1 | 4.35 |
|  |  | Lack of collaboration interest | 1 | 4.35 |
|  |  | Lack of leadership | 1 | 4.35 |
|  |  | Don’t know | 1 | 4.35 |
|  | Q16. What factors could facilitate better One Health collaboration? | Meetings/Workshops/Political action | 5 | 21.74 |
|  |  | Technical training | 4 | 17.39 |
|  |  | Communication | 3 | 13.04 |
|  |  | Professional associations | 2 | 8.70 |
|  |  | Staff availability | 1 | 4.35 |
|  |  | Negotiation processes | 1 | 4.35 |
|  |  | Don’t know | 2 | 8.70 |
