# Supplementary material for: Antimicrobial resistance in Ecuador: A One Health situational analysis of governance, infrastructure, and equity gaps
Source: PLOS Glob Public Health. 2026 Aug 21;6(8):e0005308. doi: 10.1371/journal.pgph.0005308 (PMC13496403; doi:10.1371/journal.pgph.0005308)
Supplement: Supplementary file 4 [file pgph.0005308.s003.docx]

**S3 Table. Representative verbatim quotes by analytical domain, institutional sector, and academic English translation**

| **Analytical domain** | **OH file code** | **One Health domain / Sector** | **Verbatim quote (Spanish – original)** | **Academic English translation** |
| --- | --- | --- | --- | --- |
| Intersectoral governance analysis | OH-001 | Human health | “Dentro del papel los estatutos está normado y está puesto que Una Salud es un eje transversal, pero en el momento de operativizar eso no está tan bien desarrollado y existen una serie de nudos críticos que limitan esa respuesta.” | “At the regulatory level, One Health is formally established as a cross-cutting approach; however, when it comes to implementation, it is not well developed, and there are several critical bottlenecks that limit an effective response.” |
| Intersectoral governance analysis | OH-006 | Academia | “Se intentó implementar en el 2019 un acuerdo interministerial para articular acciones, pero lamentablemente no logró instaurarse un comité que coordine de manera efectiva.” | “An attempt was made in 2019 to implement an interministerial agreement to coordinate actions; however, it ultimately failed to establish a committee capable of effective coordination.” |
| Situational analysis | OH-001 | Human health | “Estamos trabajando con cooperación técnica para llegar a un análisis situacional; ese análisis va a servir como punto de partida para poder implementar el plan.” | “We are working with technical cooperation partners to develop a situational analysis, which will serve as the starting point for implementing the plan.” |
| Situational analysis | OH-020 | Animal health | “No hemos hecho un análisis situacional que tenga en cuenta estas variables; aquí no está previsto todavía un análisis de este tipo.” | “We have not conducted a situational analysis that takes these variables into account; this type of analysis is not yet planned.” |
| Transition toward One Health | OH-015 | Civil society / Professional associations | “La colaboración y la interrelación interinstitucional son claves para entender un problema tan complejo como la resistencia a los antibióticos y avanzar hacia una salud global.” | “Collaboration and interinstitutional interaction are key to understanding a problem as complex as antimicrobial resistance and to moving toward a global health approach.” |
| Transition toward One Health | OH-018 | Civil society / Professional associations | “Una salud implica una coordinación eficaz entre actores del sector público, privado, academia y sociedad civil para lograr una salud óptima de personas, animales y ambiente.” | “One Health entails effective coordination among public and private sectors, academia, and civil society in order to achieve optimal health for people, animals, and the environment.” |
| Equity analysis using a GBA+ lens | OH-001 | Human health | “Las consideraciones de género no se integran en los programas de resistencia a los antimicrobianos; lamentablemente no se integran.” | “Gender considerations are not integrated into antimicrobial resistance programs; unfortunately, they are not incorporated.” |
| Equity analysis using a GBA+ lens | OH-020 | Animal health | “No conozco ningún estudio que tenga una perspectiva de género en una salud; de vulnerabilidad tal vez, pero de género no.” | “I am not aware of any study that applies a gender perspective within One Health; vulnerability may be considered, but gender is not.” |
