# Supplementary material for: Antimicrobial resistance in Ecuador: A One Health situational analysis of governance, infrastructure, and equity gaps
Source: PLOS Glob Public Health. 2026 Aug 21;6(8):e0005308. doi: 10.1371/journal.pgph.0005308 (PMC13496403; doi:10.1371/journal.pgph.0005308)
Supplement: Supplementary file 5 [file pgph.0005308.s004.docx]

S1 Checklist. Standards for Reporting Qualitative Research (SRQR) checklist

| SRQR Item | Topic | Description | Reported in the manuscript (section) | Status |
| --- | --- | --- | --- | --- |
| 1 | Title | Concise description of quantitative approach and topic | Title | OK |
| 2 | Abstract | Summary of background, methods, results, and conclusion | Abstract | OK |
| 3 | Problem formulation | Description of problem and rationale | Introduction | OK |
| 4 | Purpose or research question | Study objectives | Introduction | OK |
| 5 | Qualitative approach | Desing and rationale | Material and Methods – Study Design | OK |
| 6 | Researcher reflexivity | Research characteristic and positioning | Not explicitly described | Not Applicable |
| 7 | Context | Setting or contextual factors | Study Design | OK |
| 8 | Sampling strategy | How data sources were selected | Document Analysis; Interviews | OK |
| 9 | Ethical issues | Ethics approval and consent | Semi-structured interviews | OK |
| 10 | Data collection | Methods used to collect data | Materials and Methods | OK |
| 11 | Data collection instruments | Interview guide, document sources | Materials and Methods | OK |
| 12 | Units of study | Participants and documents | Table 1 | OK |
| 13 | Data processing | Transcription and coding | Data analysis | OK |
| 14 | Data analysis | Analytical procedures | Analytical Framework | OK |
| 15 | Trustworthiness | Techniques such as triangulation | Results | OK |
| 16 | Synthesis | Integration of findings | Discussion | OK |
| 17 | Link to data | Empirical support for findings | Tables 2-4; S1-S5 | Ok |
| 18 | Integration with literature | Comparison with prior work | Discussion | OK |
| 19 | Limitations | Study limitations | Discussion | OK |
| 20 | Conclusions | Implications of findings | Conclusions | OK |
| 21 | Funding | Sources of funding | Funding statement | Ok |
| 22 | Conflicts of interest | Competing interests | Competing interests | OK |
|  |  |  |  |  |
