# Supplementary material for: Antimicrobial resistance in Ecuador: A One Health situational analysis of governance, infrastructure, and equity gaps
Source: PLOS Glob Public Health. 2026 Aug 21;6(8):e0005308. doi: 10.1371/journal.pgph.0005308 (PMC13496403; doi:10.1371/journal.pgph.0005308)
Supplement: Supplementary file 6 [file pgph.0005308.s005.docx]

**Results of Thematic Coding of AMR-Related Documents**

| **Node** | **Files*** | **References**** | **Document Types** |
| --- | --- | --- | --- |
| **Governance Analysis** | 100 | 288 | 25 government documents, 11 legal regulations, 38 institutional websites, 7 journal articles, 6 newspaper articles, 1 online article, 5 books, 3 edited volumes, 4 theses. |
| *International Collaboration* | 38 | 60 | 7 government documents, 2 legal regulations, 17 institutional websites, 3 journal articles, 4 newspaper articles, 1 online article, 2 books, 1 edited volume, 1 thesis. |
| *Example of Collaborative, Interdisciplinary, or Multisectoral One Health Project* | 9 | 30 | 2 government documents, 1 institutional website, 1 journal article, 3 newspaper articles, 1 online article, 1 edited volume. |
| *Implicit One Health* | 19 | 36 | 4 government documents, 2 legal regulations, 5 institutional websites, 1 journal article, 1 newspaper article, 1 online article, 2 books, 3 edited volumes. |
| *Coordination and Multisectoral Collaboration Infrastructure* | 76 | 148 | 25 government documents, 8 legal regulations, 28 institutional websites, 4 journal articles, 2 newspaper articles, 5 books, 4 theses. |
| Intersectoral Committees | 9 | 12 | 2 government documents, 1 legal regulation, 3 institutional websites, 2 journal articles, 1 thesis. |
| Intersectoral Communication | 53 | 76 | 21 government documents, 5 legal regulations, 20 institutional websites, 2 journal articles, 1 newspaper article, 3 books, 1 thesis. |
| Coordinated Action Plans | 25 | 39 | 6 government documents, 3 legal regulations, 11 institutional websites, 1 journal article, 1 newspaper article, 1 book, 2 theses. |
| **Situational Analysis** | 90 | 293 | 17 government documents, 14 legal regulations, 37 institutional websites, 5 journal articles, 6 newspaper articles, 1 online article, 3 books, 2 edited volumes, 5 theses. |
| *Key Stakeholders* | 82 | 201 | 15 government documents, 14 legal regulations, 34 institutional websites, 6 journal articles, 3 newspaper articles, 1 online article, 3 books, 2 edited volumes, 4 theses. |
| *Mandate* | 35 | 63 | 6 government documents, 13 legal regulations, 13 institutional websites, 1 journal article, 1 newspaper article, 1 thesis. |
| Political Positions | 37 | 50 | 3 government documents, 8 legal regulations, 20 institutional websites, 2 journal articles, 1 newspaper article, 1 online article, 1 book, 1 thesis. |
| Policy Objectives | 39 | 66 | 12 government documents, 4 legal regulations, 15 institutional websites, 2 journal articles, 1 online article, 2 books, 3 theses. |
| **Transition Toward One Health** | 33 | 71 | 7 government documents, 4 legal regulations, 8 institutional websites, 3 journal articles, 3 newspaper articles, 1 online article, 1 book, 3 edited volumes, 3 theses. |
| *Barriers to Intersectoral Coordination and Collaboration* | 19 | 42 | 2 government documents, 3 institutional websites, 3 journal articles, 3 newspaper articles, 1 online article, 1 book, 3 edited volumes, 3 theses. |
| *Facilitators of Intersectoral Coordination and Collaboration* | 20 | 28 | 6 government documents, 4 legal regulations, 5 institutional websites, 2 newspaper articles, 1 online article, 2 edited volumes, 1 thesis. |
| **Gender-Based Analysis (GBA+)** | 24 | 77 | 5 government documents, 6 legal regulations, 6 journal articles, 2 books, 2 edited volumes, 3 theses. |
| *Access to Resources* | 4 | 7 | 1 government document, 1 legal regulation, 1 journal article, 1 thesis. |
| *Social Context of Equity-Deserving Groups* | 14 | 40 | 3 government documents, 1 legal regulation, 5 journal articles, 1 book, 2 edited volumes, 2 theses. |
| *Sex- and Gender-Disaggregated Data* | 2 | 2 | 1 journal article, 1 edited volume. |
| *Gender Equity in Policy* | 11 | 23 | 3 government documents, 6 legal regulations, 1 journal article, 1 book. |
| *Equity-Deserving or Vulnerable Populations* | 1 | 2 | 1 journal article. |
| *Unintended and Unexpected Equity Impacts* | 2 | 3 | 1 journal article, 1 thesis. |

* **Files**: Total number of documents coded under one or more nodes.

** **References**: Number of coded excerpts. Multiple references may appear within a single document.
