# Supplementary material for: Antimicrobial resistance in Ecuador: A One Health situational analysis of governance, infrastructure, and equity gaps
Source: PLOS Glob Public Health. 2026 Aug 21;6(8):e0005308. doi: 10.1371/journal.pgph.0005308 (PMC13496403; doi:10.1371/journal.pgph.0005308)
Supplement: Supplementary file 7 [file pgph.0005308.s007.docx]

**Response to Reviewers**

Manuscript ID:	PGPH-D-25-02802

Title:	Antimicrobial resistance in Ecuador: A One Health situational analysis of governance, infrastructure, and equity gaps

Journal:	PLOS Global Public Health

Dear Dr. Julianne Meisner

We would like to thank the Academic Editor and reviewers for their careful evaluation of our manuscript and for their constructive and insightful comments. We have revised the manuscript thoroughly, and we believe that these changes have substantially strengthened the clarity, rigor, and contribution of the study. Below, we provide a point-by-point response to each comment. All changes in the manuscript are indicated using track changes in the revised version.

For transparency, we would like to disclose that artificial intelligence tools were used exclusively to support language editing and translation from Spanish to English. No AI tools were used for study design, data analysis, interpretation of findings, or generation of scientific content. All methodological decisions, analysis, and conclusions remain entirely the responsibility of the authors. We would appreciate the Editor’s guidance on whether and where this disclosure be formally included in the manuscript.

**Academic editor and reviewers: general comments**

We sincerely thank the Academic Editor and reviewers for the careful assessment of our manuscript and for the constructive and insightful guidance provided. In response to the comments received, we have undertaken a substantial revision to ensure full alignment with PLOS Global Public Health’ publication criteria and to strengthen the methodological transparency, analytical coherence, and evidentiary grounding of the study.

The manuscript has been revised to more clearly present this work as a qualitative governance and policy analysis. The introduction now provides a concise description of Ecuador’s National Plan for the Prevention and Control of Antimicrobial Resistance (2019-2023) and clarifies the scope of the study, explicitly distinguishing it from epidemiological or microbial assessment.

We strengthened the reporting of our qualitative methodology by aligning the Methods section with the Standards for Reporting Qualitative Research (SRQR) and including a completed SRQR checklist as Supporting Information (S3 File). The Methods section now provides a transparent description of the qualitative design, data sources, analytical framework, and triangulation strategy. We clarify that no statistical analyses were conducted, as the study is based exclusively on qualitative methods.

The Results and Discussion sections have been reorganized around the four analytical domains guiding the study -intersectoral governance analysis, situational analysis, transition toward One Health, and equity analysis using a GBA+ lens- to improve coherence, readability, and analytical transparency. We expanded and clarified the presentation of findings from the document analysis, explicitly reporting emergent themes, patterns, and illustrative examples, and detailing how these were triangulated with semi-structured interview data. Throughout the manuscript, we strengthened signposting between empirical findings and interpretative claims and revised the Conclusion to ensure that all statements are directly supported by the reported data.

In response to concerns related to data availability, we revised the Data Availability Statement to full comply with PLOS data policy. While full interview transcripts and audio recordings cannot be made publicly available due to ethical and confidentiality constraints, we now provide extensive de-identified qualitative materials as Supporting Information, including thematic coding outputs from document analysis (S1 Table), summarized and anonymized interview findings (S2 Table), representative verbatim quotes (S4 Table), and the complete NVivo coding framework (S5 File). These materials provide transparent access to the data underlying the study’s findings.

Finally, we revised the Financial Disclosure statement for clarity and completeness, clarified funding sources and authors roles, removed supplementary materials from the main manuscript file, and prepared all figures and Supporting Information files in accordance with PLOS technical requirements. Minor language and stylistic revision were made throughout to ensure clarity and consistency with standard academic English.

**Reviewer # 1 – General comments**

**Comment:**

General positive assessment and recommendation to strengthen analytical clarity and empirical grounding.

**Response:**

We sincerely thanks Reviewer #1 for the positive and thoughtful assessment of our manuscript, and for recognizing its relevance to regional and global discussions on AMR governance from a One Health (OH) perspective.

In response to reviewer’s general observation, we undertook a systematic revision to strengthen analytical clarity and empirical grounding. Specifically, we improved the explicit linkage between qualitative findings (documents analysis and semi-structured interviews) and the interpretative claims presented in the Discussion and Conclusions. Signposting has been reinforced throughout the Results and Discussion to ensure the statements related to governance performance, implementation constraints, and system-level challenges are clearly grounded in the reported data.

We also clarified more explicitly that the study constitutes a qualitative governance and policy analysis rather than an epidemiological or microbiological assessment. This clarification has been reinforced in the Introduction, Methods, and Discussion sections.

In addition, we expanded the reporting of findings from the document analysis, presenting clearer descriptions of emergent themes and illustrating how these were triangulated with interview data. Supporting analytical materials -including anonymized coding outputs, interview summaries, representative verbatim quotes, and the NVivo coding framework- are now provided as Supporting Information.

We believe that these revisions substantially strengthen the methodological rigor and analytical coherence of the manuscript.

**Major comment 1**

Reviewer comments: *“While the conclusions are generally consistent with the qualitative findings, some claims, particularity those related to macro level political shifts, austerity policies, and governance deterioration, would benefit from clearer and more explicit linkage to the empirical data presented. In several instances, the discussion moves toward a normative or interpretative tone that appears to draw as much from secondary literature as from the study`s primary data. Strengthening signposting between interview findings, document analysis, and specific conclusions would improve analytical clarity”*

**Response**

We thank the reviewer for this important comment and agree that clearer linkage to empirical was needed in some instances.

As noted in our response to the general comments above, we have revised the Results, Discussion, and Conclusions sections to strengthen analytical signposting between primary qualitative data and interpretive claims. We now clearly distinguish between:

1. Findings derived directly from document analysis and interview data,
2. Stakeholder-reported perceptions, and
3. Contextual background supported by secondary literature.

Claims related to macro-level political or economic shifts are now explicitly identified as either document-supported, informant-reported, or contextual. The Conclusions have been revised to ensure that statement regarding governance deterioration, institutional weakening, and implementation constraints are proportionately grounded in the study’s empirical findings.

The revisions reduce normative tone and improve analytical transparency.

**Major comment 2**

Reviewer comments: *“The manuscript would benefit from more explicit clarification that the study is a qualitative governance and policy analysis rather than an epidemiological assessment of antimicrobial resistance trends. Readers may otherwise expect microbiological or quantitative AMR indicators, which are outside the scope of this work but not always clearly distinguished in the framing”.*

**Response**

We thank the reviewer for this helpful observation.

As indicated in our response to the general comments we have revised the framing of the manuscript to clearly and consistently position the study as a qualitative governance and policy analysis. The Introduction now explicitly state that the study does not assess AMR prevalence, microbiological patterns, or quantitative indicators, but instead examines institutional arrangements, intersectoral coordination, infrastructure, and equity dimensions of the national AMR response.

The Methods section more clearly describe the qualitative design and analytical approach, and the Discussion and Conclusions have been adjusted to avoid any implication of epidemiological inference.

These revisions clarify the scope of the study and align reader expectations with its objectives.

**Major comment 3**

Reviewer comments: *“The Data Availability Statements indicates that all relevant data are included within the manuscript, and the additional information is available upon seasonable request. However, this does not fully meet PLOS data policy requirements. The primary qualitative data underlying the findings, such as anonymized interview transcripts, coded data excerpts or NVivo codebooks, are not publicly available as supplementary files or deposited in a repository. If there are ethical or confidentiality constraints that prevent public sharing of these materials, these restrictions should be clearly specified in the Data Availability Statement. Alternatively, the authors are encouraged to share de-identified qualitative data, coding frameworks, or analytic matrices as Supporting Information to enhance transparency and reproducibility”.*

**Response**

We thank the reviewer for this important comment.

As noted in the general response above, we have revised the Data Availability Statement to fully comply with PLOS data policy. Ethical and confidentiality preventing public release of full interview transcripts and audio recordings are now clearly specified.

To enhance transparency while respecting ethical approvals, we provide extensive de-identified qualitative materials as Supporting Information including:

1. Thematic coding outputs from the documents analysis (S1 Table),
2. Anonymized interviews findings organized by analytical domains (S2 Table),
3. Representative anonymized verbatim quotes (S4 Table),
4. The complete NVivo coding framework (S5 File); and
5. The SRQR checklist (S3 File).

These materials ensure transparent access to qualitative evidence underlying the study’s findings while maintaining confidentially protections.

**Minor comment (a)**

Reviewer comments: *“Consider minor language and stylistic revisions throughout the manuscript to improve clarity and flow, particularly in the Introduction and Discussion sections”.*

**Response**

We have revised the manuscript for clarity, and consistency, with particular attention to the Introduction and Discussion sections.

**Minor comment (b)**

Reviewer comments: *“Ensure consistent terminology when referring to governance structures, committees, and surveillance systems”.*

**Response**

Terminology has been standardized throughout the manuscript to ensure consistent to governance structures, committees, and surveillance systems.

**Minor comment (c)**

Reviewer comments: *“Some tables (e.g., interview results) could benefit from brief interpretive summaries to guide readers unfamiliar with the Ecuadorian institutional context”.*

**Response**

Brief interpretive summaries have been added to relevant tables to improve contextual understanding.

**Minor comment (d)**

Reviewer comments: *“The equity analysis is a strong component of the manuscript; however, explicitly distinguishing between findings derived from interview data versus documents analysis would further strengthen this section”.*

**Response**

As also addressed in Major Comment 1, we have revised the equity analysis to explicitly differentiate findings derived from interview from those based on document analysis.

**Reviewer # 2 – General comments**

**Comment:**

Broader claims were not always supported by sufficiently explicit reporting of document-analysis findings; need stronger integration and visibility of documentary evidence.

**Response:**

We thank Reviewer #2 for the careful evaluation and constructive overview.

We agree with the original manuscript did not sufficiently foreground findings from document analysis despite this being a central component of the study. In response, we substantially expanded the Results section to present clearer description emergent themes, patterns, gaps and illustrative examples identified through systematic document analysis. We also strengthened the explicit integration and triangulation of documentary findings with interview data across the Results and Discussion.

As detailed further in Major Comment 3 below, these revisions enhance empirical transparency and analytical coherence.

**Major comment 1**

Reviewer comments: *“The Introduction would benefit from a brief description of Ecuador’s National Plan for the prevention and Control of AMR (2019-2023) – including its overarching goals, structure, key components/strategic axes, and intended governance/implementation approach. This context is necessary for readers to understand what was constrained in implementation and to interpret the claims made in the Discussion and Conclusions”.*

**Response**

We thank the reviewer for this important suggestion.

We revised the Introduction to include as concise but explicit description of the National AMR Plan, outlining its overarching goals, governance and implementation structure, strategic axes, and intended multisectoral coordination mechanism. We also clarified the role of National AMR Committee in overseeing implementation.

This contextual addition strengthens readers’ understanding of the institutional framework within which the identified implementation constraints are situated.

**Major comment 2**

Reviewer comments: *“The Methods section needs substantial revision to clearly describe how the qualitative research was conducted and analyzed. I recommend aligning the reporting with SRQR (Standards for Reporting Qualitative Research) and citing: O'Brien BC, Harris IB, Beckman TJ, Reed DA, Cook DA. Standards for reporting qualitative research: a synthesis of recommendations. Academic Medicine. 2014;89(9):1245-1251. Please consider including an SRQR checklist as Supplementary Information to improve transparency and reproducibility of the qualitative analysis”*

**Response**

We agree that the Methods section required clearly reporting.

The section has been comprehensively revised to align the reporting with the Standards for Reporting Qualitative Research (SRQR), now providing a structured description of study design, sampling recruitment, data source, data collection, data management, and analytical procedures, including document analysis and semi-structured interview components, including the development of the interview guide, data triangulation, and thematic coding supported by NVivo.

O’Brien et al. (2014) is now explicitly cited as the guiding reporting framework, and a completed SRQR checklist has been including as Supporting Information (S3 File).

These revisions substantially strengthen the methodological transparency and rigor.

**Major comment 3**

Reviewer comments: *“The manuscript currently provides limited explicit reporting of findings from the document analysis, despite this being a central component of the study. Please present clearer, more detailed results from the document analysis (e.g., what patterns/themes emerged, concrete examples), and explain how these findings were integrated with (or triangulated against) the semi-structured interview data”.*

**Response**

We thank the reviewer for this critical observation.

As noted in the General Comments above, the Results section has been substantially expanded to explicitly report patterns, themes, and gaps identified accross 117 policy, regulatory, institutional, and scientific documents. Findings are organized according to the four analytical domains guiding the study and include illustrative examples and quantitative descriptors of the coded material.

We now explicitly indicate whether findings derive from documentary sources, interview data, or their convergence, thereby clarifying triangulation.

Detailed document-analysis outputs -including thematic coding matrices and the NVivo coding framework- are provided as Supporting Information (S1 Table and S5 File).

**Major comment 4**

Reviewer comments: *“As written, the Results and Discussion sections are difficult to follow. Consider restructuring the manuscript around the four analytical themes/framework domains used in the study: 1. Intersectoral governance analysis; 2. Situational analysis; 3. Transitions toward One Health; 4. Equity analysis using a GBA+ lens. Using these as consistent subheadings throughout would strengthen coherence and readability”.*

**Response**

We agree and have restructured both the Results and Discussion sections using the four analytical domains as explicit and consistent subheadings:

1. Intersectoral governance analysis
2. Situational analysis
3. Transition toward One Health
4. Equity analysis using a GBA+ lens.

This reorganization improves coherence, logical flow, and transparency between empirical findings and interpretation.

**Major comment 5**

Reviewer comments: *“The Discussion does not yet fully unpack what the findings mean, nor does it adequately situate them in relation to experiences from other countries (Latin America, LMIC settings, and high-income settings where implementation has been more effective or similarly constrained). Additionally, the manuscript states that it proposes a "context-specific action framework," but this framework is not clearly presented or easy to locate. If this is a key contribution ("So what? What now?"), please make it explicit”.*

**Response**

We agree that interpretative depth required strengthening.

The Discussion has been substantially expanded to more explicitly interpret the implications of findings for AMR governance under a One Health approach and to situate Ecuador’s experience with Latin America, LMIC, and selected high-income contexts.

The proposed “context-specific action framework” is now presented in a clearly labeled subsection within the Discussion and visually summarized in a dedicated figure (Figure 2). This makes the manuscript’s practical contribution explicit and directly addresses the “so what” and “what now” questions.

**Major comment 6**

Reviewer comments: *“Several conclusions currently extend beyond what is clearly supported by the Results section. Please ensure the Conclusions are tightly grounded in the reported evidence (from both document analysis and interviews), or revise/soften claims where direct supporting data are not presented”.*

**Response**

We agree and have carefully revised the Conclusions to ensure strict alignment with reported findings.

Statements regarding governance capacity, information system interoperability, laboratory infrastructure, and resources constraints are now explicitly attributed to triangulated findings from document analysis and interview data. Where financial or structural changes were not directly documented, language has been calibrated to reflect stakeholder-reported perceptions rather than empirically verified longitudinal budget analysis.

These revisions reduce overinterpretation and strengthen the internal coherence.

**Minor comment (a)**

Reviewer comments: *“Line 46: Please briefly define selective pressure and explain how it contributes to the emergence and spread of antimicrobial-resistant microorganisms”.*

**Response**

We have added a brief definition (Lines ~52-55).

**Minor comment (b)**

Reviewer comments: *“Line 59: Before discussing constrains, briefly describe the Ecuadorian National PLAN for the Prevention and Control of AMR (2019-2023)”.*

**Response**

Addressed in Major Comments 1 (Introduction revised).

**Minor comment (c)**

Reviewer comments: *“Line 72: Grammar: “reduced” instead of “reduces”.*

**Response**

Corrected.

**Minor comment (d)**

Reviewer comments: *“Lines 75-78: Clarify whether statements are derived from data or literature; add references in contextual”.*

**Response**

Revised to explicitly signpost data sources (Lines ~77-78)

**Minor comment (e)**

Reviewer comments: *“Line 75: Consider starting a new paragraph around here to introduce the National Plan/Committee context more clearly before transitioning into limitations”.*

**Response**

New paragraph introduced to improve narrative flow (Lines ~66-75)

**Minor comment (f)**

Reviewer comments: *“Line 106: Please briefly define semi-structured interviews and include a reference for the approach”.*

**Response**

Definition and references added in Methods (Lines ~140-144)

**Minor comment (g)**

Reviewer comments: *“Lines 106-108: The study objective is already stated earlier; considering removing repeated objective language here to streamline the Methods”.*

**Response**

Redundant statement removed.

**Minor comment (h)**

Reviewer comments: *“Recommended adding a clearly labeled Ethics subsection (IRB approval/waiver, consent procedures, confidentiality protections)”.*

**Response**

Dedicated “Ethics considerations” subsection added (Lines ~146-152)

**Minor comment (i)**

Reviewer comments: *“Lines 150-151, 157-158, 160-161: These appear related and could be consolidated into one coherent paragraph to improve flow”.*

**Response**

Interview procedures and analysis consolidated into one coherent paragraph (Lines ~197-203).

**Minor comment (j)**

Reviewer comments: *“Table 1: Please provide more detail on the “affiliated agencies”/ “agencies” included. For example, within the Ministry of Health, does this include INSPI or other specific bodies? Consider organizing the table using headings aligned with your interview sampling frame (e.g., human health, animal health, environment, academia, civil society) to match the manuscript text”.*

**Response**

Table reorganized according to One Health domains, institutional affiliations clarified.

**Minor comment (k)**

Reviewer comments: *“Line 173: Please define the acronym GBA+ at first use”.*

**Response**

Defined at first occurrence.

**Minor comment (l)**

Reviewer comments: *“The Methods section would benefit from clearer subsections. Suggested structure:1. Study design and setting; 2. Sampling and participants (sampling strategy, eligibility criteria, recruitment, number approached/interviewed; how you determined sampling adequacy/saturation); 3.Data sources and data collection document analysis: document types, inclusion criteria, extraction approach interviews: interview guide development, interviewer training/positionality if relevant, interview mode, audio recording, transcription/translation, any iterative changes to guides; 4. Data management (storage/security, deidentification/anonymization, coding workflow); 5. Data analysis (analytic approach for documents and interviews; how themes were developed; triangulation across methods; reflexivity/rigor strategies such as audit trail, double coding, member checking if used)”.*

**Response**

Methods organized in line with SRQR guidance (see Major Comments 2).

**Minor comment (m)**

Reviewer comments: *“Consider including (as Supplementary Information) an SRQR checklist to improve transparency Results”.*

**Response**

Included as supplementary information (S3 File)

**Minor comment (n)**

Reviewer comments: *“The Results section would be clearer if organized explicitly around your four analytical themes/framework domains: 1. Intersectoral governance analysis; 2. Situational analysis; 3. Transitions toward One Health; 4. Equity analysis using a GBA+ lens. Consider using these as subheadings and presenting findings under each”.*

**Response**

Addressed in Major Comment 4.

**Minor comment (ñ)**

Reviewer comments: *“There is currently little explicit reporting of what was found from the document analysis. Please include concrete results from that component (e.g., what patterns/gaps were identified, and specific examples)”.*

**Response**

Addressed in Major Comment 3.

**Minor comment (o)**

Reviewer comments: *“Lines 207–212: This reads like interpretation more appropriate for the Discussion (and would likely need supporting references if it’s a broader claim). Consider moving it”.*

**Response**

Results revised to retain descriptive findings only, interpretative material relocated to Discussion.

**Minor comment (p)**

Reviewer comments: *“Lines 223–226: These statements also appear interpretive and would fit better in the Discussion”.*

**Response**

Results revised to retain descriptive findings only, interpretative material relocated to Discussion.

**Minor comment (q)**

Reviewer comments: *“Line 228: “Barriers and facilitators” are introduced here but not clearly set up earlier. If identifying barriers/facilitators is a central objective, please introduce it in the Introduction/Aims and ensure consistent framing throughout”.*

**Response**

Introduction revised for consistent framing (Lines ~101-104).

**Minor comment (r)**

Reviewer comments: *“Lines 234–241; 243–249: These sections read like discussion/interpretation rather than results. Consider revising to focus on what participants/documents explicitly reported (with evidence) and move broader implications to the Discussion”.*

**Response**

Results revised to retain descriptive findings only, interpretative material relocated to Discussion.

**Minor comment (s)**

Reviewer comments: *“Consider adding a small number of representative verbatim quotes from the semi-structured interviews to support each major theme. Including 1–2 quotes per theme (with anonymized participant identifiers/roles) would strengthen credibility and transparency and is standard for reporting semi-structured interview findings. If space is limited, quotes can be placed in a table or supplement”.*

**Response**

Representative anonymized quotes added as Supporting Information (S4 Table).

**Minor comment (t)**

Reviewer comments: *“Consider organizing the Discussion using the same four analytical themes as the Results to improve coherence and readability”.*

**Response**

Addressed in Major Comment 4

**Minor comment (u)**

Reviewer comments: *“The Discussion would benefit from deeper comparison with related work from Latin America and other LMIC settings, as well as contrasting with experiences in high-income settings where national AMR plans may have been implemented more effectively. This would strengthen interpretation and generalizability”.*

**Response**

Addressed in Major Comment 5

**Minor comment (v)**

Reviewer comments: *“The Introduction indicates that a “context-specific action framework” is proposed; however, this is not easy to locate in the current manuscript. Please clearly identify where the framework is presented (potentially Lines 320–327?) and consider adding a figure/table or a clearly labeled subsection so readers can easily find and understand it”.*

**Response**

Addressed in Major Comment 5 (dedicated subsection + Figure 2)

**Minor comment (w)**

Reviewer comments: *“• Overall, the conclusions are plausible, but some claims appear stronger than what is currently supported by the Results section, especially without clearly presented document-analysis findings.*

*• For example, the statement about deterioration in governance capacities, information system interoperability, laboratory infrastructure, and budget allocations would be strengthened by explicit evidence from the document analysis and/or interviews. If budget shifts were assessed, please report what sources were used and what changes were observed; if not directly assessed, consider softening the language or clarifying that it reflects stakeholder perceptions rather than documented budgetary evidence”.*

**Response**

Addressed in Major Comment 6.

**Final Comments**

We sincerely thank the Academic Editor and the reviewers for the time, expertise, and constructive feedback invested in the evaluation of our manuscript. We believe that the revisions undertaken in response to their comments have substantially strengthened the methodological rigor, analytical clarity, transparency, and overall contribution of the study.

The revised manuscript now presents a clearer and more coherent qualitative governance and policy analysis and antimicrobial resistance in Ecuador under a One Health framework. Key improvements include enhance reporting and triangulation of documents analysis and interview findings, explicit alignment with SRQR reporting standards, improved organization of the Results and Discussion sections around the analytical framework, and clearer grounding of conclusions in the empirical evidence.

We hope that the revised manuscript adequately addressed all concerns raised and meets the publication standards of **PLOS Global Public Health.** We are grateful the opportunity to revise and resubmit our work and would be pleases to provide any additional clarifications if required.

Kind regards,

Richar Rodríguez-Hidalgo, PhD

On behalf of all authors
