# Supplementary material for: Antimicrobial resistance in Ecuador: A One Health situational analysis of governance, infrastructure, and equity gaps
Source: PLOS Glob Public Health. 2026 Aug 21;6(8):e0005308. doi: 10.1371/journal.pgph.0005308 (PMC13496403; doi:10.1371/journal.pgph.0005308)
Supplement: Peer review history

# Peer review: Decision Letter 0

**DOI:** 10.1371/journal.pgph.0005308.r001

**Authors:** Julianne Meisner

8 Jan 2026

PGPH-D-25-02802

Antimicrobial resistance in Ecuador: A One Health situational analysis of governance, infrastructure, and equity gaps

PLOS Global Public Health

Dear Dr. Rodríguez-Hidalgo,

Thank you for submitting your manuscript to PLOS Global Public Health. After careful consideration, we feel that it has merit but does not fully meet PLOS Global Public Health’s publication criteria as it currently stands. Therefore, we invite you to submit a revised version of the manuscript that addresses the points raised during the review process.

==============================

Please be sure to address each reviewer's comment in turn

==============================

Please submit your revised manuscript by Feb 22 2026 11:59PM. If you will need more time than this to complete your revisions, please reply to this message or contact the journal office at globalpubhealth@plos.org. When you're ready to submit your revision, log on to https://www.editorialmanager.com/pgph/ and select the 'Submissions Needing Revision' folder to locate your manuscript file.

Please include the following items when submitting your revised manuscript:

- A letter that responds to each point raised by the editor and reviewer(s). You should upload this letter as a separate file labeled 'Response to Reviewers'.
- A marked-up copy of your manuscript that highlights changes made to the original version. You should upload this as a separate file labeled 'Revised Manuscript with Track Changes'.
- An unmarked version of your revised paper without tracked changes. You should upload this as a separate file labeled 'Manuscript'.

Guidelines for resubmitting your figure files are available below the reviewer comments at the end of this letter.

We look forward to receiving your revised manuscript.

Kind regards,

Julianne Meisner

Academic Editor

PLOS Global Public Health

Journal Requirements:

1. Please include a complete copy of PLOS’ questionnaire on inclusivity in global research in your revised manuscript. Our policy for research in this area aims to improve transparency in the reporting of research performed outside of researchers’ own country or community. The policy applies to researchers who have travelled to a different country to conduct research, research with Indigenous populations or their lands, and research on cultural artefacts. The questionnaire can also be requested at the journal’s discretion for any other submissions, even if these conditions are not met. Please find more information on the policy and a link to download a blank copy of the questionnaire here: https://journals.plos.org/globalpublichealth/s/best-practices-in-research-reporting. Please upload a completed version of your questionnaire as Supporting Information when you resubmit your manuscript.

2. Please amend your detailed Financial Disclosure statement. This is published with the article. It must therefore be completed in full sentences and contain the exact wording you wish to be published.

1. Please clarify all sources of funding (financial or material support) for your study. List the grants (with grant number) or organizations (with url) that supported your study, including funding received from your institution.

2. State the initials, alongside each funding source, of each author to receive each grant.

3. State what role the funders took in the study. If the funders had no role in your study, please state: “The funders had no role in study design, data collection and analysis, decision to publish, or preparation of the manuscript.”

4. If any authors received a salary from any of your funders, please state which authors and which funders.

3. Please provide separate figure files in .tif or .eps format.

For more information about figure files please see our guidelines:

https://journals.plos.org/globalpublichealth/s/figures

https://journals.plos.org/globalpublichealth/s/figures#loc-file-requirements

4. We notice that your supplementary [figures/tables] are included in the manuscript file. Please remove them and upload them with the file type 'Supporting Information'. Please ensure that each Supporting Information file has a legend listed in the manuscript after the references list.

5. In the online submission form, you indicated that All relevant data supporting the findings of this study are fully available within the manuscript. Aditional information is available from the authors upon reasonable request.

All PLOS journals now require all data underlying the findings described in their manuscript to be freely available to other researchers, either

1. In a public repository,

2. Within the manuscript itself, or

3. Uploaded as supplementary information.

This policy applies to all data except where public deposition would breach compliance with the protocol approved by your research ethics board. If your data cannot be made publicly available for ethical or legal reasons (e.g., public availability would compromise patient privacy), please explain your reasons by return email and your exemption request will be escalated to the editor for approval. Your exemption request will be handled independently and will not hold up the peer review process, but will need to be resolved should your manuscript be accepted for publication. One of the Editorial team will then be in touch if there are any issues.

If the reviewer comments include a recommendation to cite specific previously published works, please review and evaluate these publications to determine whether they are relevant and should be cited. There is no requirement to cite these works unless the editor has indicated otherwise.

Additional Editor Comments:

Please address each reviewer's comment in turn

[Note: HTML markup is below. Please do not edit.]

Reviewers' comments:

Reviewer's Responses to Questions

**Comments to the Author**

1. Does this manuscript meet PLOS Global Public Health’s publication criteria?

Reviewer #1: Partly

Reviewer #2: No

**********

2. Has the statistical analysis been performed appropriately and rigorously?

Reviewer #1: Yes

Reviewer #2: N/A

**********

3. Have the authors made all data underlying the findings in their manuscript fully available (please refer to the Data Availability Statement at the start of the manuscript PDF file)?

The PLOS Data policy

Reviewer #1: No

Reviewer #2: No

**********

4. Is the manuscript presented in an intelligible fashion and written in standard English?

Reviewer #1: Yes

Reviewer #2: Yes

**********

Reviewer #1: This manuscript addresses antimicrobial resistance in Ecuador through a One Health lens, focusing on governance, infrastructure, and equity. The topic is highly relevant to PLOS Global Public Health, particularly given the emphasis on health systems, intersectoral governance, and equity in low and middle income country contexts. The study makes a valuable contribution to regional and global discussions on AMR governance.

Some points need to be addressed:

1. While the conclusions are generally consistent with the qualitative findings, some claims, particularly those related to macro level political shifts, austerity policies, and governance deterioration, would benefit from clearer and more explicit linkage to the empirical data presented. In several instances, the discussion moves toward a normative or interpretive tone that appears to draw as much from secondary literature as from the study’s primary data. Strengthening signposting between interview findings, document analysis, and specific conclusions would improve analytical clarity.

2. The manuscript would benefit from more explicit clarification that the study is a qualitative governance and policy analysis rather than an epidemiological assessment of antimicrobial resistance trends. Readers may otherwise expect microbiological or quantitative AMR indicators, which are outside the scope of this work but not always clearly distinguished in the framing.

3. The Data Availability Statement indicates that all relevant data are included within the manuscript and that additional information is available upon reasonable request. However, this does not fully meet PLOS data policy requirements. The primary qualitative data underlying the findings, such as anonymized interview transcripts, coded data excerpts, or NVivo codebooks, are not publicly available as supplementary files or deposited in a repository.

If there are ethical or confidentiality constraints that prevent public sharing of these materials, these restrictions should be clearly specified in the Data Availability Statement. Alternatively, the authors are encouraged to share de-identified qualitative data, coding frameworks, or analytic matrices as Supporting Information to enhance transparency and reproducibility.

Here minnor suggestions:

a) Consider minor language and stylistic revisions throughout the manuscript to improve clarity and flow, particularly in the Introduction and Discussion sections.

b) Ensure consistent terminology when referring to governance structures, committees, and surveillance systems.

c) Some tables (e.g., interview results) could benefit from brief interpretive summaries to guide readers unfamiliar with the Ecuadorian institutional context.

The equity analysis is a strong component of the manuscript; however, explicitly distinguishing between findings derived from interview data versus document analysis would further strengthen this section.

Reviewer #2: Overview:

This study examines national approaches to addressing antimicrobial resistance (AMR) in Ecuador from a One Health (OH) perspective, with emphasis on governance, public policy, health infrastructure, and equity. The authors use a qualitative design combining document review, scientific literature analysis, and semi-structured interviews with key informants representing multiple OH sectors. The manuscript offers a useful overview of the challenges Ecuador faces in implementing an OH approach to AMR prevention.

However, many of the broader claims are not sufficiently supported by the evidence currently presented. In particular, findings from the document analysis, the central component of the study, are not reported in a clear or substantive way, making it difficult to assess how the conclusions were derived. Strengthening the presentation of document-analysis results, clarifying how these findings were integrated with interview data, and improving the organization and flow of the manuscript would substantially increase its rigor and impact. With these revisions, the paper has the potential to become a valuable contribution to the literature on AMR and One Health in Ecuador.

Major revisions

• The Introduction would benefit from a brief description of Ecuador's National Plan for the Prevention and Control of AMR (2019-2023)-including its overarching goals, structure, key components/strategic axes, and intended governance/implementation approach. This context is necessary for readers to understand what was constrained in implementation and to interpret the claims made in the Discussion and Conclusions.

• The Methods section needs substantial revision to clearly describe how the qualitative research was conducted and analyzed. I recommend aligning the reporting with SRQR (Standards for Reporting Qualitative Research) and citing: O'Brien BC, Harris IB, Beckman TJ, Reed DA, Cook DA. Standards for reporting qualitative research: a synthesis of recommendations. Academic Medicine. 2014;89(9):1245-1251. Please consider including an SRQR checklist as Supplementary Information to improve transparency and reproducibility of the qualitative analysis.

• The manuscript currently provides limited explicit reporting of findings from the document analysis, despite this being a central component of the study. Please present clearer, more detailed results from the document analysis (e.g., what patterns/themes emerged, concrete examples), and explain how these findings were integrated with (or triangulated against) the semi-structured interview data.

• As written, the Results and Discussion sections are difficult to follow. Consider restructuring the manuscript around the four analytical themes/framework domains used in the study: 1. Intersectoral governance analysis; 2. Situational analysis; 3. Transitions toward One Health; 4. Equity analysis using a GBA+ lens. Using these as consistent subheadings throughout would strengthen coherence and readability.

• The Discussion does not yet fully unpack what the findings mean, nor does it adequately situate them in relation to experiences from other countries (Latin America, LMIC settings, and high-income settings where implementation has been more effective or similarly constrained). Additionally, the manuscript states that it proposes a "context-specific action framework," but this framework is not clearly presented or easy to locate. If this is a key contribution ("So what? What now?"), please make it explicit.

• Several conclusions currently extend beyond what is clearly supported by the Results section. Please ensure the Conclusions are tightly grounded in the reported evidence (from both document analysis and interviews), or revise/soften claims where direct supporting data are not presented.

Minor revisions

Introduction

• Line 46: Please briefly define selective pressure and explain how it contributes to the emergence and spread of antimicrobial-resistant microorganisms.

• Line 59: Before discussing constraints, it would help to briefly describe the Ecuadorian National Plan for the Prevention and Control of AMR (2019–2023), for example, its overarching goals, structure, key components/strategic axes, and intended governance/implementation approach. This context will help readers understand what specific aspects were constrained.

• Line 72: Grammar: “reduced” instead of “reduces.”

• Lines 75–78: These statements read as interpretive claims; please clarify whether they are based on cited literature or derived from your data. If they are claims about broader context, references are needed.

• Line 75: Consider starting a new paragraph around here to introduce the National Plan/Committee context more clearly before transitioning into limitations.

Methods

• Line 106: Please briefly define semi-structured interviews and include a reference for the approach.

• Lines 106–108: The study objective is already stated earlier; consider removing repeated objective language here to streamline the Methods.

• Recommend adding a clearly labeled Ethics subsection (IRB approval/waiver, consent procedures, confidentiality protections).

• Lines 150–151, 157–158, 160–161: These appear related and could be consolidated into one coherent paragraph to improve flow.

• Table 1: Please provide more detail on the “affiliated agencies”/ “agencies” included. For example, within the Ministry of Health, does this include INSPI or other specific bodies? Consider organizing the table using headings aligned with your interview sampling frame (e.g., human health, animal health, environment, academia, civil society) to match the manuscript text.

• Line 173: Please define the acronym GBA+ at first use.

The Methods section would benefit from clearer subsections. Suggested structure:1. Study design and setting; 2. Sampling and participants (sampling strategy, eligibility criteria, recruitment, number approached/interviewed; how you determined sampling adequacy/saturation); 3.Data sources and data collection document analysis: document types, inclusion criteria, extraction approach interviews: interview guide development, interviewer training/positionality if relevant, interview mode, audio recording, transcription/translation, any iterative changes to guides; 4. Data management (storage/security, de-identification/anonymization, coding workflow); 5. Data analysis (analytic approach for documents and interviews; how themes were developed; triangulation across methods; reflexivity/rigor strategies such as audit trail, double coding, member checking if used).

• Consider including (as Supplementary Information) an SRQR checklist to improve transparency

Results

• The Results section would be clearer if organized explicitly around your four analytical themes/framework domains: 1. Intersectoral governance analysis; 2. Situational analysis; 3. Transitions toward One Health; 4. Equity analysis using a GBA+ lens. Consider using these as subheadings and presenting findings under each.

• There is currently little explicit reporting of what was found from the document analysis. Please include concrete results from that component (e.g., what patterns/gaps were identified, and specific examples).

• Lines 207–212: This reads like interpretation more appropriate for the Discussion (and would likely need supporting references if it’s a broader claim). Consider moving it.

• Lines 223–226: These statements also appear interpretive and would fit better in the Discussion.

• Line 228: “Barriers and facilitators” are introduced here but not clearly set up earlier. If identifying barriers/facilitators is a central objective, please introduce it in the Introduction/Aims and ensure consistent framing throughout.

• Lines 234–241; 243–249: These sections read like discussion/interpretation rather than results. Consider revising to focus on what participants/documents explicitly reported (with evidence) and move broader implications to the Discussion.

• Consider adding a small number of representative verbatim quotes from the semi-structured interviews to support each major theme. Including 1–2 quotes per theme (with anonymized participant identifiers/roles) would strengthen credibility and transparency and is standard for reporting semi-structured interview findings. If space is limited, quotes can be placed in a table or supplement.

Discussion

• Consider organizing the Discussion using the same four analytical themes as the Results to improve coherence and readability.

• The Discussion would benefit from deeper comparison with related work from Latin America and other LMIC settings, as well as contrasting with experiences in high-income settings where national AMR plans may have been implemented more effectively. This would strengthen interpretation and generalizability.

• The Introduction indicates that a “context-specific action framework” is proposed; however, this is not easy to locate in the current manuscript. Please clearly identify where the framework is presented (potentially Lines 320–327?) and consider adding a figure/table or a clearly labeled subsection so readers can easily find and understand it.

Conclusion

• Overall, the conclusions are plausible, but some claims appear stronger than what is currently supported by the Results section, especially without clearly presented document-analysis findings.

• For example, the statement about deterioration in governance capacities, information system interoperability, laboratory infrastructure, and budget allocations would be strengthened by explicit evidence from the document analysis and/or interviews. If budget shifts were assessed, please report what sources were used and what changes were observed; if not directly assessed, consider softening the language or clarifying that it reflects stakeholder perceptions rather than documented budgetary evidence.

**********

what does this mean?). If published, this will include your full peer review and any attached files.

**Do you want your identity to be public for this peer review?**  If you choose “no”, your identity will remain anonymous but your review may still be made public.

For information about this choice, including consent withdrawal, please see our Privacy Policy

Reviewer #1: **Yes:** Maria de Jesus Medina Arellano

Reviewer #2: No

**********

[NOTE: If reviewer comments were submitted as an attachment file, they will be attached to this email and accessible via the submission site. Please log into your account, locate the manuscript record, and check for the action link "View Attachments". If this link does not appear, there are no attachment files.]

# Author comment: Author response to Decision Letter 1

**DOI:** 10.1371/journal.pgph.0005308.r002

17 Feb 2026

Attachment Submitted filename: Response to Reviewers.docx

# Peer review: Decision Letter 1

**DOI:** 10.1371/journal.pgph.0005308.r003

**Authors:** Sarah Jose

11 May 2026

PGPH-D-25-02802R1

Antimicrobial resistance in Ecuador: A One Health situational analysis of governance, infrastructure, and equity gaps

PLOS Global Public Health

Dear Dr. Rodríguez-Hidalgo,

Thank you for submitting your manuscript to PLOS Global Public Health. After careful consideration, we feel that it has merit but does not fully meet PLOS Global Public Health’s publication criteria as it currently stands. Therefore, we invite you to submit a revised version of the manuscript that addresses the points raised during the review process.

Please review the feedback from the reviewers and revise your manuscript accordingly, providing a point-by-point response to the reviewers upon resubmission. Please ensure that all citations are correctly reported in your References section and that the text is still accessible.

Please submit your revised manuscript by Jul 10 2026 11:59PM. If you will need more time than this to complete your revisions, please reply to this message or contact the journal office at globalpubhealth@plos.org. When you're ready to submit your revision, log on to https://www.editorialmanager.com/pgph/ and select the 'Submissions Needing Revision' folder to locate your manuscript file.

Please include the following items when submitting your revised manuscript:

- A letter that responds to each point raised by the editor and reviewer(s). You should upload this letter as a separate file labeled 'Response to Reviewers'.
- A marked-up copy of your manuscript that highlights changes made to the original version. You should upload this as a separate file labeled 'Revised Manuscript with Track Changes'.
- An unmarked version of your revised paper without tracked changes. You should upload this as a separate file labeled 'Manuscript'.

Guidelines for resubmitting your figure files are available below the reviewer comments at the end of this letter.

As the corresponding author, your ORCID iD is verified in the submission system and will appear in the published article. PLOS supports the use of ORCID, and we encourage all coauthors to register for an ORCID iD and use it as well. Please encourage your coauthors to verify their ORCID iD within the submission system before final acceptance, as unverified ORCID iDs will not appear in the published article. *Only*  the individual author can complete the verification step; PLOS staff *cannot*  verify ORCID iDs on behalf of authors.

We look forward to receiving your revised manuscript.

Kind regards,

Sarah Jose, Ph.D.

Staff Editor

PLOS Global Public Health

**Journal Requirements:**

If the reviewer comments include a recommendation to cite specific previously published works, please review and evaluate these publications to determine whether they are relevant and should be cited. There is no requirement to cite these works unless the editor has indicated otherwise.

**Additional Editor Comments (if provided):**

[Note: HTML markup is below. Please do not edit.]

Reviewers' comments:

Reviewer's Responses to Questions

**Comments to the Author**

Reviewer #1: (No Response)

Reviewer #2: (No Response)

**********

publication criteria?

Reviewer #1: Partly

Reviewer #2: (No Response)

**********

3. Has the statistical analysis been performed appropriately and rigorously?

Reviewer #1: No

Reviewer #2: (No Response)

**********

4. Have the authors made all data underlying the findings in their manuscript fully available (please refer to the Data Availability Statement at the start of the manuscript PDF file)?

The PLOS Data policy

Reviewer #1: Yes

Reviewer #2: (No Response)

**********

5. Is the manuscript presented in an intelligible fashion and written in standard English?

Reviewer #1: No

Reviewer #2: (No Response)

**********

**Reviewer #1:**  The manuscript addresses an important and timely public health issue, and the authors should be commended for the substantial improvements made in this revised version, particularly in clarifying the qualitative design and strengthening the overall structure of the paper. The study has clear potential to make a valuable contribution to the literature on AMR governance and One Health in Ecuador and the region. However, a small number of remaining editorial, consistency, and clarity issues should still be addressed before the manuscript is ready for publication.

Some of the revisions that I identify are the following ones:

1. Please, do replace all placeholder text in the figure captions and notes. Figure 1 still contains placeholder text (“This is the Fig1 Title” and “This is the Fig 1 Note”), and Figure 2 also still contains placeholder text in both the title and note. These must be replaced with the final, publication ready caption text.

2. Please, do correct the typo in Figure 2 (ARM should be AMR) and check figure numbering consistency.

The Figure 2 caption currently reads “strengthening ARM governance,” which is clearly incorrect. It also refers to “This is the Fig 1 Title,” suggesting that figure labeling was not finalized.

3. The manuscript still requires a final, careful language and copyediting revision, since there are residual grammatical and wording problems throughout the text, for example: “a preventive measures,” “have hindering,” “Search termsincluded,” “AMR polices,” “low income,” “country setting,” and “subjected to ethical approval.” A thorough proofread is still needed.

4. Please, do correct remaining terminology and spelling issues in Table 1. For example, “Enviromental health” should be “Environmental health,” and “affiliates agencies” should be revised for grammatical correctness (e.g., “affiliated agencies”).

5. Please, do further clarify the evidentiary basis of the macro political and austerity related claims. The Introduction still includes strong statements about the shift to a neoliberal model, austerity measures, budget cuts, and institutional weakening. These points may be valid, but they should be signposted more explicitly as either: (a) contextual background from secondary literature, (b) document-based findings, or (c) stakeholder-reported perceptions.

6. Please, do make the basis for budgetary and resource related claims more explicit at the point of use.

The manuscript refers to insufficient budget allocation, financial constraints, and limited human and material resources. Where these are based on documentary evidence, the relevant sources should be made explicit, where they are based mainly on stakeholder perceptions, the wording should be softened accordingly.

7. Please, you should add a brief limitations paragraph that explicitly acknowledges sectoral imbalance in the interview sample. This would strengthen the methodological transparency of the paper. In particular, environmental health appears to be represented by only one participant, which should be acknowledged as a limitation when discussing transferability across all One Health sectors.

Although the manuscript has improved substantially and I have identified few of the remaining issues that still need correction, I encourage the authors to undertake a careful and meticulous final revision of the entire manuscript. This is a valuable and timely study with clear potential to contribute meaningfully to the literature on AMR governance and One Health in Ecuador and the region. With these final corrections, the paper will be in a much stronger position for publication.

**Reviewer #2:**  Although the topic and findings are relevant for the country and the broader region, the manuscript still needs stronger analysis to support its conclusions. The organization and writing have improved, especially in how the results are presented, but the depth of analysis is still limited. The discussion, in particular, lacks critical interpretation of the findings.

In addition, several references (at least 10 out of 33) could not be accessed because the cited websites are no longer available. These seem to be important sources supporting the funding claims made by the authors. Without access to these documents, it is difficult to assess or verify how that information is being interpreted.

**********

what does this mean?). If published, this will include your full peer review and any attached files.

**Do you want your identity to be public for this peer review?**  If you choose “no”, your identity will remain anonymous but your review may still be made public.

For information about this choice, including consent withdrawal, please see our Privacy Policy

Reviewer #1: **Yes:** MARIA DE JESUS MEDINA ARELLANO

Reviewer #2: No

**********

[NOTE: If reviewer comments were submitted as an attachment file, they will be attached to this email and accessible via the submission site. Please log into your account, locate the manuscript record, and check for the action link "View Attachments". If this link does not appear, there are no attachment files.]

**Figure Resubmissions:**

# Author comment: Author response to Decision Letter 2

**DOI:** 10.1371/journal.pgph.0005308.r004

25 May 2026

Attachment Submitted filename: Response to Reviewers_R2-signed.pdf

# Editor report: Decision Letter 2

**DOI:** 10.1371/journal.pgph.0005308.r005

**Authors:** Bipin Adhikari

28 May 2026

PGPH-D-25-02802R2

Antimicrobial resistance in Ecuador: A One Health situational analysis of governance, infrastructure, and equity gaps

PLOS Global Public Health

Dear Dr. Rodríguez-Hidalgo,

Thank you for submitting your manuscript to PLOS Global Public Health. After careful consideration, we feel that it has merit but does not fully meet PLOS Global Public Health’s publication criteria as it currently stands. Therefore, we invite you to submit a revised version of the manuscript that addresses the points raised during the review process.

Please submit your revised manuscript by Jun 27 2026 11:59PM. If you will need more time than this to complete your revisions, please reply to this message or contact the journal office at globalpubhealth@plos.org. When you're ready to submit your revision, log on to https://www.editorialmanager.com/pgph/ and select the 'Submissions Needing Revision' folder to locate your manuscript file.

Please include the following items when submitting your revised manuscript:

- A letter that responds to each point raised by the editor and reviewer(s). You should upload this letter as a separate file labeled 'Response to Reviewers'.
- A marked-up copy of your manuscript that highlights changes made to the original version. You should upload this as a separate file labeled 'Revised Manuscript with Track Changes'.
- An unmarked version of your revised paper without tracked changes. You should upload this as a separate file labeled 'Manuscript'.

Guidelines for resubmitting your figure files are available below the reviewer comments at the end of this letter.

As the corresponding author, your ORCID iD is verified in the submission system and will appear in the published article. PLOS supports the use of ORCID, and we encourage all coauthors to register for an ORCID iD and use it as well. Please encourage your coauthors to verify their ORCID iD within the submission system before final acceptance, as unverified ORCID iDs will not appear in the published article. *Only*  the individual author can complete the verification step; PLOS staff *cannot*  verify ORCID iDs on behalf of authors.

We look forward to receiving your revised manuscript.

Kind regards,

Bipin Adhikari, MBBS, DTM&H, MCTM, MPH, DPhil

Academic Editor

PLOS Global Public Health

Journal Requirements:

If the reviewer comments include a recommendation to cite specific previously published works, please review and evaluate these publications to determine whether they are relevant and should be cited. There is no requirement to cite these works unless the editor has indicated otherwise.

Please review your reference list to ensure that it is complete and correct. If you have cited papers that have been retracted, please include the rationale for doing so in the manuscript text, or remove these references and replace them with relevant current references. Any changes to the reference list should be mentioned in the rebuttal letter that accompanies your revised manuscript. If you need to cite a retracted article, indicate the article’s retracted status in the References list and also include a citation and full reference for the retraction notice.

Additional Editor Comments (if provided):

This is an important study and is highly relevant to current discourse on One Health, including the challenges posed by increasing integrated approaches to tackling AMR. I have seen the manuscript undergo peer review, and thus I am reviewing it in my editorial capacity to strengthen its standing.

Here are broad suggestions for the authors to improve the manuscript:

1. In the Methods section, clearly state or reference the One Health framework. Alternatively, add a line on One Health framework and cite a reference.

2. Line 127: The study design was intended to examine…… Did you have any existing theoretical framework guiding your analysis? There are theoretical frameworks applied to One Health in AMR you might want to build on: https://gh.bmj.com/content/10/10/e019236

3. Add a line on why you chose these specific countries: Canada, Brazil, Rwanda, and Ecuador.

4. Analytical framework and data analysis: Your second thematic analysis clearly presents some elements of stakeholder analysis. Within this section or elsewhere in the methods, clearly mention how you picked these elements (stakeholder roles, institutional positions, priorities…), among others.

5. Equity analysis using a gender-based analysis — have you got a reference to back this analysis?

6. A lot of paragraphs within the discussion section could be engaged with more extensive literature from your own document analysis. Consider discussing the literature, whether and how your findings differ or echo with others.

7. Line 42: While the relevance… This is a valid point. Again, relate/discuss this with similar literature.

8. Line 505: Our findings suggest that limited… This is again an important point; discuss this with the literature, whether this is consistent across other geographical regions.

9. And then the following paragraphs as well; consider engaging with more literature.

10. Reduce your conclusion section to a paragraph (you could turn it into sets of recommendations), as it repeats much of what you have already reported in previous sections.

[Note: HTML markup is below. Please do not edit.]

Reviewers' comments:

[NOTE: If reviewer comments were submitted as an attachment file, they will be attached to this email and accessible via the submission site. Please log into your account, locate the manuscript record, and check for the action link "View Attachments". If this link does not appear, there are no attachment files.]

# Author comment: Author response to Decision Letter 3

**DOI:** 10.1371/journal.pgph.0005308.r006

10 Jun 2026

Attachment Submitted filename: Response to Reviewers_R3-signed.pdf

# Editor report: Decision Letter 3

**DOI:** 10.1371/journal.pgph.0005308.r007

**Authors:** Bipin Adhikari

12 Jun 2026

Antimicrobial resistance in Ecuador: A One Health situational analysis of governance, infrastructure, and equity gaps

PGPH-D-25-02802R3

Dear Dr. Rodríguez-Hidalgo,

We are pleased to inform you that your manuscript 'Antimicrobial resistance in Ecuador: A One Health situational analysis of governance, infrastructure, and equity gaps' has been provisionally accepted for publication in PLOS Global Public Health.

Before your manuscript can be formally accepted you will need to complete some formatting changes, which you will receive in a follow up email. A member of our team will be in touch with a set of requests.

Please note that your manuscript will not be scheduled for publication until you have made the required changes, so a swift response is appreciated.

IMPORTANT: The editorial review process is now complete. PLOS will only permit corrections to spelling, formatting or significant scientific errors from this point onwards. Requests for major changes, or any which affect the scientific understanding of your work, will cause delays to the publication date of your manuscript.

If your institution or institutions have a press office, please notify them about your upcoming paper to help maximize its impact. If they'll be preparing press materials, please inform our press team as soon as possible -- no later than 48 hours after receiving the formal acceptance. Your manuscript will remain under strict press embargo until 2 pm Eastern Time on the date of publication. For more information, please contact globalpubhealth@plos.org.

Thank you again for supporting Open Access publishing; we are looking forward to publishing your work in PLOS Global Public Health.

Best regards,

Bipin Adhikari, MBBS, DTM&H, MCTM, MPH, DPhil

Academic Editor

PLOS Global Public Health

***********************************************************

Reviewer Comments (if any, and for reference):
